# Effect of the *ortho*-hydroxy group of salicylaldehyde in the A^3^ coupling reaction: A metal-catalyst-free synthesis of propargylamine

**DOI:** 10.3762/bjoc.13.53

**Published:** 2017-03-16

**Authors:** Sujit Ghosh, Kinkar Biswas, Suchandra Bhattacharya, Pranab Ghosh, Basudeb Basu

**Affiliations:** 1 Department of Chemistry, University of North Bengal, Darjeeling 734013, India

**Keywords:** A^3^ coupling, metal-catalyst-free, propargylamine, salicylaldehyde, terminal alkyne

## Abstract

The synthesis of propargylamines via A^3^ coupling mostly under metal-catalyzed procedures is well known. This work invented an unprecedented effect of salicylaldehyde, one of the A^3^ coupling partners, which could lead to the formation of propargylamine, an important pharmaceutical building block, in the absence of any metal catalyst and under mild conditions. The role of the hydroxy group in *ortho* position of salicylaldehyde has been explored, which presumably activates the C_sp_–H bond of the terminal alkyne leading to the formation of propargylamines in good to excellent yields, thus negating the function of the metal catalyst. This observation is hitherto unknown, tested for a variety of salicylaldehyde, amine and acetylene, established as a general protocol, and is believed to be of interest for synthetic chemists from green chemistry.

## Introduction

**Scheme 1:**
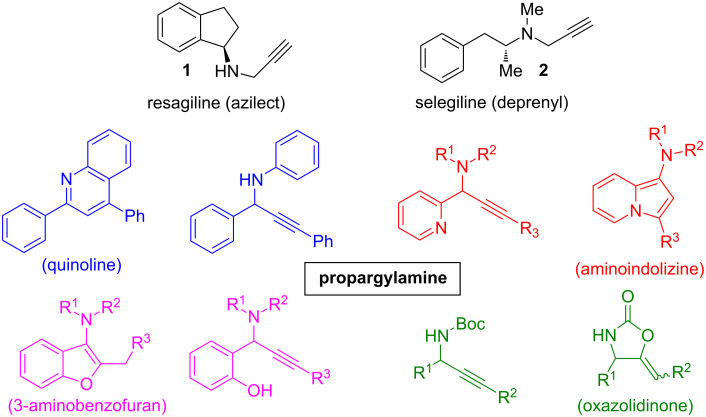
Representative examples of bioactive compounds bearing a propargylamine moiety and synthesis of various *N*-heterocycles from propargylamine-containing intermediates.

Propargylamines are important synthetic intermediates for the preparation of natural products [1], potential therapeutic agents [2], oxotremorine analogues [3] and multifunctional amino derivatives [4–5]. Compounds like resagiline or selegiline (structures **1** and **2**, Scheme 1) bearing a propargylamine moiety, are familiar as potent selective, irreversible monoamine oxidase (MAO) type B inhibitors [6] often used for the treatment of neuropsychiatric disorders such as Alzheimer’s and Parkinson diseases. These alkynylamines are also important building blocks for the synthesis of *N*-bearing compounds such as β-lactams [7–8], pyrroles [9], pyrrolidines [10], pyrrolophanes [11], 3-aminobenzofurans [12], aminoindolizines [13], 2-aminoimidazoles [14], oxazolidinones [15], and quinolines [16] (Scheme 1).

**Scheme 2:**
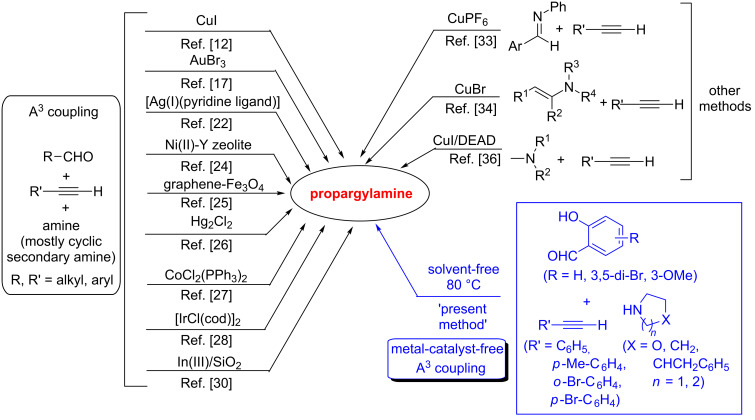
Various metal-catalyzed methods for the synthesis of propargylamine.

Because of diverse applications of propargylamine, several methods are developed among which the three–component reaction of aldehyde, amine and terminal alkyne, commonly known as A^3^ coupling reaction, remains the most common and straightforward method for the synthesis of propargylamine. The A^3^ coupling reaction is reported under transition-metal-catalyzed conditions using copper [12,17–19], gold [17,20–21], silver [17,22], zinc [17,23], nickel [24], iron [25], mercury [26], cobalt [27], iridium [28], ruthenium [29], indium [30] etc. Other methods towards the synthesis of propargylamine include: alkynylation of imine [31–33], enamine [34], and C_sp³_–H bonds adjacent to *N*-atoms [35–36]. In the A^3^ coupling, the role of the metal catalyst is believed to activate the terminal acetylene primarily, which then undergoes a nucleophilic addition to the iminium electrophile generated from the aldehyde and the amine. Among different transition metals, copper metal has been mostly explored as the catalyst to activate the terminal acetylene, though there is a possibility for the Glaser coupling of the terminal alkyne as the byproduct [37]. Interestingly, hitherto there is no report for the A^3^ coupling reaction in the absence of a metal catalyst except of one example in a three-component reaction using an alkynylcarboxylic acid instead of a terminal alkyne [38]. In this case, activation of the C_sp_–COOH occurs via decarboxylation followed by the coupling with an iminium electrophile to produce the propargylamine. Although the strategy is interesting, functionalized acetylene carboxylic acids are difficultly accessible and the reaction is less 'atom economic'. Therefore, the development of a metal-free and straightforward greener protocol for the preparation of propargylamine is highly desirable. A schematic presentation of various metal-catalyzed protocols including the present work is outlined in Scheme 2.

## Results and Discussion

In connection with our interest in developing new organic reaction methodologies as well as to synthesize propargylamine, we performed one reaction of salicylaldehyde, morpholine and phenylacetylene at 80 °C in the presence of a Cu(I) dithiane-based complex as the catalyst that gave rise to the formation of **3a** in good yield (89%, Table 1, entry 1). To check the role of our Cu(I) catalyst, we performed the same reaction under similar conditions in the absence of the copper catalyst (Table 1, entry 2). To our surprise, the same product spot was noticed on TLC plate and further work-up and purification afforded the product in comparable isolated yield. We became interested about our findings and wanted to explore the reaction further in the absence of a metal catalyst. We did the same experiment with *o*-anisaldehyde and 2-chlorobenzaldehyde without using any copper catalyst, and interestingly, there was no desired product formed in these two reactions (Table 1, entries 3 and 4). Conducting the same experiment with *p*-hydroxybenzaldehyde (Table 1, entry 5) or *o*-hydroxyacetopheneone (Table 1, entry 6) also did not generate the corresponding A^3^ coupled product.

**Table 1:** Optimization of metal-catalyst-free A^3^ coupling reaction.

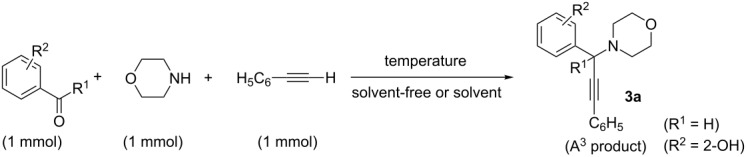						

Entry	R^1^	R^2^	Temperature (°C)	Time (h)	Solvent	Yield (%)^a^

1^b^	H	2-OH	80	4	neat	89
2	H	2-OH	80	4	neat	90
3	H	2-OMe	80	4	neat	no reaction
4	H	2-Cl	80	4	neat	no reaction
5	H	4-OH	80	4	neat	no reaction
6	Me	2-OH	80	4	neat	no reaction
7	H	2-OH	rt	24	neat	traces (<10%)
8	H	2-OH	60	10	neat	64
9^c^	H	2-OH	80	4	MeCN	85
10^c^	H	2-OH	80	4	toluene	88
11^c^	H	2-OH	80	4	EtOH	47
12^d^	H	2-OH	80	10	neat	no A^3^ product, only imine formation
13^e^	H	2-OH	80	10	neat	no A^3^ product, only imine formation

^a^Yield of product after purification by column chromatography; ^b^using a mixture of CuI and 1,3-dithiane ligand [1-(3-(*p*-tolylthio)propylthio)-4-methylbenzene] in 1:2 ratios (1 mol %); ^c^2 mL of solvent was taken; ^d^cyclohexylamine (1 mmol) was used instead of morpholine; ^e^benzylamine (1 mmol) was used instead of morpholine.

The above observations made us curious about any specific role of the hydroxy group in the *ortho* position of salicylaldehyde in A^3^ coupling. A search in the literature revealed vast examples of A^3^ coupling with different benzaldehydes. However, only few research groups [12,19–24] reported their experiments with salicylaldehyde only in the presence of metal catalysts (e.g., Cu, Au, Ag, Zn and Ni). We therefore explored further to optimize the reaction conditions in the absence of Cu catalysts at varying temperatures and solvents. In the optimization process (Table 1), we found that the reaction was very slow at room temperature even after 24 h (<10%; Table 1, entry 7), and raising the reaction temperature to 60 °C afforded an improved yield of the product (64%; Table 1, entry 8). We carried out the reaction in solvents like acetonitrile (Table 1, entry 9), toluene (Table 1, entry 10) and ethanol (Table 1, entry 11). Although high yields were achieved in the first two solvents (85–88%), the use of ethanol resulted in a low yield (47%). Thus the A^3^ coupling of salicylaldehyde, phenylacetylene and morpholine can be achieved under solvent-free conditions as well as in solvents like toluene or acetonitrile with the formation of the coupled product in 85–90% isolated yields. We also conducted similar reactions with cyclohexylamine and benzylamine instead of morpholine. In both cases, we ended up with the formation of corresponding imines (Table 1, entries 12 and 13).

**Figure 1:**
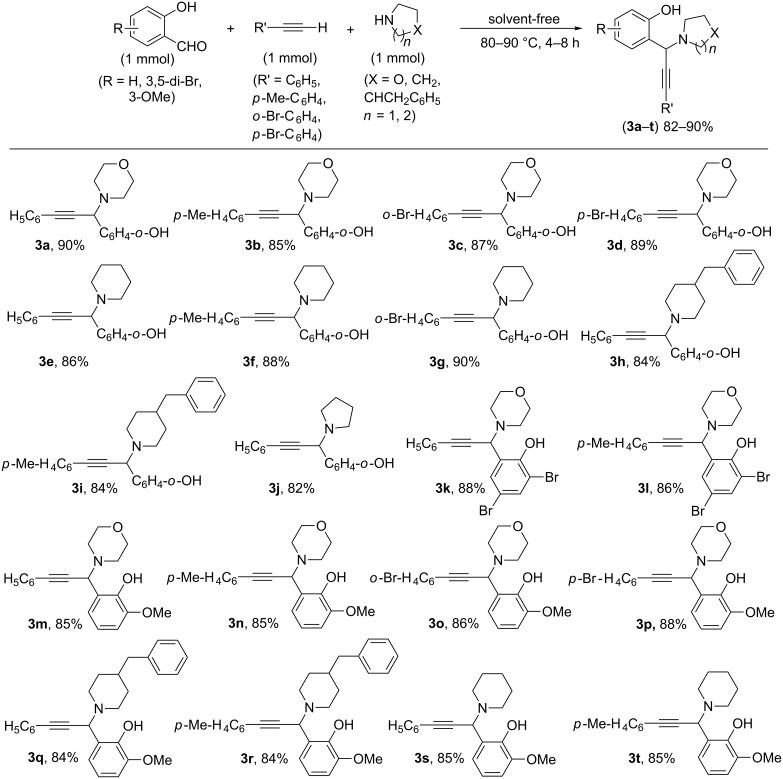
Synthesis of various propargylamines from various salicylaldehydes under metal-catalyst-free conditions^.^

After the successful optimization, further extension of the reaction protocol was made by varying the other two components viz. the secondary cyclic amine and the terminal alkyne. The results are not only encouraging but constitute a hitherto unknown general protocol for the preparation of propargylamines under metal-catalyst-free A^3^ coupling of salicylaldehyde as the aldehyde component (Figure 1). Changing the phenylacetylene to other substituted arylacetylenes like *p*-tolylacetylene, *o*-bromophenylacetylene, *p*-bromophenylacetylene or switching from morpholine to other amines like piperidine, 4-benzylpiperidine and pyrrolidine also react smoothly to afford the corresponding propargylamine (the A^3^ coupled product) in 82–90% isolated yields (Figure 1, **3a–j**). Substituted salicylaldehyde like 3,5-dibromosalicylaldehyde or *o*-vanillin also reacted easily when treated with morpholine and phenylacetylene, *p*-tolylacetylene or *o*- and *p*-bromophenylacetylene as the other coupling components at 80–90 °C (Figure 1, **3k–p**). Varying the secondary amine component with 4-benzylpiperidine, piperidine also worked efficiently to produce the corresponding propargylamine derivatives (**3q–t**). All products were characterized by FTIR, ^1^H and ^13^C NMR spectral data. Scaling up the reaction to gram-scale operation using phenylacetylene, morpholine and salicylaldehyde (5 mmol each) also afforded the desired propargylamine cleanly in 86% yield.

A mixture of salicylaldehyde or its derivative (1 mmol), amine (1 mmol) and alkyne (1 mmol) was stirred at 80 °C (90 °C for dibromo derivative of salicylaldehyde) in a sealed tube for 4–8 h (4 h for **3a–l** and 8 h for **3m–t**). Isolated yields after purification by column chromatography are given.

### Mechanism

**Scheme 3:**
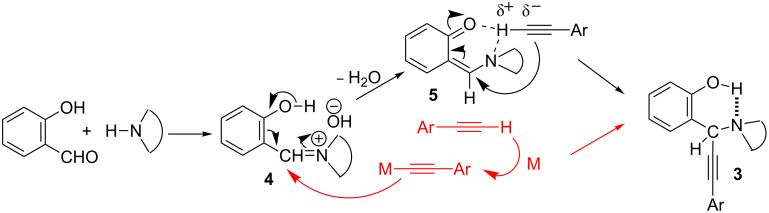
Plausible mechanism for the metal-free A^3^ coupling from salicylaldehyde.

As regard to the mechanism of the reaction, it is generally believed that the metal catalyst activates the terminal alkyne so as to generate metal acetylide species, which then undergoes nucleophilic addition at the electrophilic iminium ion **4**, eventually providing the A^3^ coupled product (Scheme 3). In the absence of the metal catalyst, the sp carbon (C–H) of the alkyne is less nucleophilic in nature. However, after the formation of the iminium ion **4** (as it happens in all such cases), the hydroxy group in the *ortho* position may undergo deprotonation forming an unstable *o*-quinonoid intermediate **5**, which presumably activates the sp carbon (C–H) of the alkyne more nucleophilic via H-bond formation to **5**, and finally to form the more stable A^3^ coupled product **3**. Although we are not sure about the exact routes to the formation of **3**, other results indirectly corroborate the proposition. For example, the inability of the *o*-methoxybenzaldehyde, *p*-hydroxybenzaldehyde and *o*–hydroxyacetophenone to undergo a similar reaction under metal−free conditions could be explained by this mechanism. The *p*-hydroxybenzaldehyde might give rise to the corresponding *p*-quinonoid species but not activates the sp carbon (C–H) of the alkyne. Again, in the case of *o*-hydroxyacetophenone, the corresponding iminium salt might form with difficulty as well as the iminium carbon would be less elctrophilic. Furthermore, the reaction does not proceed well in a protic solvent like ethanol. This suggests that the hydroxy group of salicylaldehyde can also make the hydrogen bonding with protic ethanol solvent and thereby reducing the possibility of activating the sp carbon (C–H) of the alkyne. Furthermore, reactions with primary amines like cyclohexylamine and benzylamine produce only the imine and no A^3^-coupled product, signifying that imines are less efficient than iminium species to initiate further reaction with terminal alkyne.

## Conclusion

In conclusion, the present study demonstrates an unusual but added role of the hydroxy group of salicylaldehyde, which paves the way to develop metal-free as well as solvent-free A^3^ coupling reactions leading to the formation of propargylamine – the useful synthetic intermediate and an important unit of many bio-active compounds. The reaction conditions are straightforward and products are obtained in good to excellent yields. The metal-free approach also offers the advantages of avoiding any possible byproduct arising out from the Glaser coupling of terminal alkynes as well as contamination with metal species. The present protocol supersedes the only metal-catalyst-free approach from acetylene carboxylic acids that are difficultly accessible and with low atom economy. Thus the present reaction from easily available A^3^ components leading to the formation of propargylamine under metal-catalyst-free and solvent-free conditions could attract the interest of synthetic and medicinal chemists.

## Supporting Information

File 1 Experimental procedure, characterization data and scan copies of ^1^H and ^13^C NMR spectra (**3a–t**).
