# Supplementary material for: Effect of the ortho-hydroxy group of salicylaldehyde in the A3 coupling reaction: A metal-catalyst-free synthesis of propargylamine
Source: Beilstein J Org Chem. 2017 Mar 16;13:552–7. doi: 10.3762/bjoc.13.53 (PMC5372705; doi:10.3762/bjoc.13.53)
Supplement: File 1 — Experimental procedure, characterization data and scan copies of 1H and 13C NMR spectra (3a–t). [file Beilstein_J_Org_Chem-13-552-s001.pdf]

Supporting Information

for

Effect of the *ortho*-hydroxy group of salicylaldehyde in the A^3 coupling reaction: A metal-catalyst-free synthesis of propargylamine

Sujit Ghosh, Kinkar Biswas, Suchandra Bhattacharya, Pranab Ghosh and Basudeb Basu*

Address: Department of Chemistry, University of North Bengal, Darjeeling 734013, India

Email: Basudeb Basu - basu_nbu@hotmail.com

*Corresponding author

Experimental procedure, characterization data and scan copies of ^1H and ^{13}C NMR spectra (3a–t)

Table of contents

I. **General information** **S2**

II. **General procedure of the A^3 coupling reaction** **S2**

III. **Characterization data of different propargylamine derivatives (3a–t)** **S2–S7**

IV. **References** **S7**

V. **Scanned spectra (^1H and ^{13}C NMR) of propargylamine derivatives (3a–t)** **S8–S47**

I. General information

o-Vanillin, phenylacetylene, *p*-tolylacetylene, *o*-bromophenylacetylene, *p*-bromophenylacetylene and 4-benzylpiperidine were purchased from Sigma−Aldrich and used directly as obtained. Salicylaldehyde was purchased from SD Fine Chemical Limited, India. Morpholine, piperidine and pyrrolidine were purchased from Lancaster and used after distillation. All the products were purified by column chromatography on 60–120 mesh silica gels (SRL, India). For TLC, Merck plates coated with silica gel 60, F_{254} were used. FTIR spectra were recorded in Perkin Elmer FTIR spectrometer using KBr pellets for solid compounds and under neat condition for liquid compounds. The peak patterns in FTIR spectra were denoted by s (sharp), m (medium), w (weak) and br (broad). The ^{1}H and ^{13}C NMR spectra were recorded at 300 MHz and 75 MHz, respectively on a Bruker AV 300 spectrometer in $CDCl_3$. Splitting patterns of protons were described as s (singlet), d (doublet), t (triplet), br (broad) and m (multiplet). Chemical shifts (δ) were reported in parts per million (ppm) relative to TMS as internal standard. *J* values (coupling constant) were reported in Hz (Hertz). ^{13}C NMR spectra were recorded with complete proton decoupling ($CDCl_3$: δ 77.0 ppm). High resolution mass spectra (HRMS) were done under ESI (positive mode) in a Micromass Q-TOF Spectrometer at Indian Association for the Cultivation of Science, Kolkata, India or in a Brucker Maxis Spectrometer at National Institute of Pharmaceutical Education and Research, Mohali, India. Melting points were determined by heating the solid sample in a sealed capillary tube taken in concentrated H_2SO_4 bath and are uncorrected.

II. General procedure of the A^3 coupling reaction

An equimolar mixture of salicylaldehyde, amine and acetylene (1 mmol of each component) was magnetically stirred (oil bath temp. 80 °C or 90 °C) in a Teflon-capped sealed tube for 4–8 h. After completion of the reaction (disappearance of the alkyne spot on TLC), the reaction mixture was cooled to room temperature, CH_2Cl_2 (2 mL) was added and adsorbed with silica gel. After evaporation of the solvent, the resulting dry mass was placed on a silica gel column and elution with light petroleum−ethyl acetate (in varying proportion) afforded the desired propargylamine.

III. Characterization data for different propargylamine derivatives (3a–t)

2-(1-Morpholino-3-phenylprop-2-ynyl)phenol,[1] (3a): Yellow solid (mp 94–96 ^{o}C); FTIR (KBr): 3414 (m, br), 3044 (s), 2970 (s), 2838 (s), 2369 (w), 1586 (s), 1487 (s), 1236 (s), 1115 (s), 755 (s), 692 (s) cm^{-1}; ^{1}H NMR ($CDCl_3$, 300 MHz): δ 2.78 (s, 4H, 2 x OCH_2), 3.80 (s, 4H, 2 x NCH_2), 5.10 (s, 1H, CH), 6.86–6.91 (m, 2H, ArH), 7.22–7.27 (m, 1H, ArH), 7.35–7.37 (m, 3H, ArH), 7.53–7.58 (m, 3H, ArH), 10.76 (s, br, 1H, OH); ^{13}C NMR ($CDCl_3$, 75 MHz): δ 47.8 (NCH_2), 59.6 (CH), 65.7 (OCH_2), 80.5 (acetylenic carbon), 89.3 (acetylenic carbon), 115.4, 118.3, 119.5, 121.2, 127.3, 127.61, 127.63, 128.6, 130.8, 155.9 (aromatic carbons).

2-(1-Morpholino-3-*p*-tolylylprop-2-ynyl)phenol (3b): Yellow solid (mp 88–90 °C); FTIR (KBr): 3415 (m, br), 2915 (s), 2846 (s), 2361 (w), 1582 (s), 1454 (s), 1278 (s), 1114 (s), 812 (s), 754 (s), 560 (s) cm^{-1}; ^{1}H NMR ($CDCl_3$, 300 MHz): δ 2.38 (s, 3H, CH_3), 2.78 (s, 4H, 2 x OCH_2), 3.80 (s, 4H, 2 x NCH_2), 5.08 (s, 1H, CH), 6.85–6.97 (m, 2H, ArH), 7.15–7.26 (m,

3H, ArH), 7.42–7.52 (m, 2H, ArH), 7.56–7.64 (m, 1H, ArH), 10.80 (s, br, 1H, OH); ^{13}C NMR ($CDCl_3$, 75 MHz): δ 21.5 (CH_3), 48.5 (NCH_2), 60.8 (CH), 66.9 (OCH_2), 80.8 (acetylenic carbon), 90.6 (acetylenic carbon), 116.5, 119.2, 119.4, 120.7, 128.8, 129.2, 129.7, 131.8, 139.0, 157.0 (aromatic carbons). HRMS (ESI) $[M+Na]^+$: calcd ($C_{20}H_{21}NO_2Na$) 330.1470, found 330.1466.

2-(3-(2-Bromophenyl)-1-morpholinoprop-2-ynyl)phenol (3c): Off-white solid (mp 102–104 ^{o}C); FTIR (KBr): 3416 (m, br), 2942 (s), 2849 (s), 2364 (w), 1584 (s), 1470 (s), 1242 (s), 1114 (s), 756 (s), 574 (s) cm^{-1}; ^{1}H NMR ($CDCl_3$, 300 MHz): δ 2.84 (s, 4H, 2 x OCH_2), 3.81 (s, 4H, 2 x NCH_2), 5.16 (s, 1H, CH), 6.86–6.92 (m, 2H, ArH), 7.19–7.34 (m, 3H, ArH), 7.57–7.67 (m, 3H, ArH), 10.74 (s, br, 1H, OH); ^{13}C NMR ($CDCl_3$, 75 MHz): δ 49.1 (NCH_2), 60.8 (CH), 66.9 (OCH_2), 86.5 (acetylenic carbon), 88.7 (acetylenic carbon), 116.5, 119.5, 120.3, 124.6, 125.6, 127.1, 128.9, 129.8, 129.9, 132.6, 133.8, 157.0 (aromatic carbons). HRMS (ESI) $[M+Na]^+$: calcd ($C_{19}H_{18}BrNO_2Na$) 394.0419, found 394.0419.

2-(3-(4-Bromophenyl)-1-morpholinoprop-2-ynyl)phenol (3d): Yellow sticky solid; FTIR (KBr): 3408 (w, br), 2842 (m), 2364 (w), 1584 (s), 1471 (s), 1270 (s), 1115 (s), 1073 (s), 999 (s), 844 (s), 746 (s), 584 (s) cm^{-1}. ^{1}H NMR ($CDCl_3$, 300 MHz): δ 2.74 (s, 4H, 2 x OCH_2), 3.77 (s, 4H, 2 x NCH_2), 5.05 (s, 1H, CH), 6.86–6.91 (m, 2H, ArH), 7.21–7.27 (m, 1H, ArH), 7.37–7.54 (m, 5H, ArH), 10.69 (s, 1H, br, OH). ^{13}C NMR ($CDCl_3$, 75 MHz): δ 48.6 (NCH_2), 60.4 (CH), 66.5 (OCH_2), 82.7 (acetylenic carbon), 89.0 (acetylenic carbon), 116.3, 119.2, 120.0, 120.9, 122.8, 128.4, 129.6, 131.4, 133.0, 156.7 (aromatic carbons). HRMS (ESI) $[M+Na]^+$: calcd ($C_{19}H_{18}BrNO_2Na$) 394.0419, found 394.0421.

2-(3-Phenyl-1-(piperidin-1-yl)prop-2-ynyl)phenol,[2] (3e): Yellow liquid; FTIR (Neat): 3055 (w), 2936 (s), 2853 (s), 2221 (w), 1692 (s) 1590 (s), 1464 (s), 1277 (s), 1108 (s), 1084 (s), 974 (s), 754 (s), 691 (s), 670 (s), 543 (s) cm^{-1}; ^{1}H NMR ($CDCl_3$, 300 MHz): δ 1.52 (s, br, 2H, $N(CH_2)_2\underline{CH_2}$), 1.68 (s, 4H, 2 x $NCH_2\underline{CH_2}$), 2.72 (t, J = 5.1 Hz, 4H, 2 x NCH_2), 5.09 (s, 1H, CH), 6.83–6.88 (m, 2H, ArH), 7.20–7.25 (m, 1H, ArH), 7.34–7.38 (m, 3H, ArH), 7.52–7.57 (m, 3H, ArH); ^{13}C NMR ($CDCl_3$, 75 MHz): δ 24.0 ($N(CH_2)_2\underline{C}H_2$), 26.0 ($NCH_2\underline{C}H_2$), 50.0 ($NCH_2$), 61.1 (CH), 82.4 (acetylenic carbon), 89.8 (acetylenic carbon), 116.4, 119.0, 121.3, 122.7, 128.42, 128.45, 128.6, 129.4, 131.9, 157.7 (aromatic carbons). HRMS (ESI) $[M+H]^+$: calcd ($C_{20}H_{22}NO$) 292.1701, found 292.1693.

2-(1-(Piperidin-1-yl)-3-*p*-tolylprop-2-ynyl)phenol (3f): Yellow viscous liquid; FTIR (Neat): 3416 (m, br), 3028 (s), 2935 (s), 2854 (s), 2830 (s), 2361 (w), 2217 (w), 1690 (s), 1607 (s), 1586 (s), 1509 (s), 1465 (s), 1278 (s), 1084 (s), 976 (s), 816 (s), 754 (s), 530 (s) cm^{-1}; ^{1}H NMR ($CDCl_3$, 300 MHz): δ 1.59 (s, br, 2H, $N(CH_2)_2\underline{CH_2}$), 1.75 (s, 4H, 2 x $NCH_2\underline{CH_2}$), 2.44 (s, 3H, CH_3), 2.79 (s, 4H, 2 x NCH_2), 5.15 (s, 1H, CH), 6.91–6.96 (m, 2H, ArH), 7.22–7.32 (m, 3H, ArH), 7.53 (d, J = 8.07 Hz, 2H, ArH), 7.64–7.68 (m, 1H, ArH); ^{13}C NMR ($CDCl_3$, 75 MHz): δ 21.6 (CH_3), 24.1 ($N(CH_2)_2\underline{C}H_2$), 26.1 ($NCH_2\underline{C}H_2$), 49.8 ($NCH_2$), 61.2 (CH), 81.7 (acetylenic carbon), 90.0 (acetylenic carbon), 116.4, 119.1, 119.6, 121.5, 128.6, 129.2, 129.4, 131.9, 138.8, 157.8 (aromatic carbons). HRMS (ESI) $[M+H]^+$: calcd ($C_{21}H_{24}NO$) 306.1858, found 306.1854.

2-(3-(2-Bromophenyl)-1-(piperidin-1-yl)prop-2-ynyl)phenol (3g): Pale yellow liquid; FTIR (Neat): 3054 (s), 2936 (s), 2862 (s), 2223 (w), 1690 (s), 1592 (s), 1466 (s), 1243 (s), 1156 (s), 1083 (s), 973 (s), 751 (s), 675 (s), 560 (s) cm^{-1}; ^{1}H NMR ($CDCl_3$, 300 MHz): δ 1.52 (s, br, 2H, $N(CH_2)_2\underline{CH_2}$), 1.68 (s, 4H, 2 x $NCH_2\underline{CH_2}$), 2.78 (s, 4H, 2 x NCH_2), 5.15 (s, 1H, CH), 6.84–6.88 (m, 2H, ArH), 7.18–7.34 (m, 3H, ArH), 7.56–7.65 (m, 3H, ArH); ^{13}C NMR ($CDCl_3$, 75 MHz): δ 24.0 ($N(CH_2)_2\underline{C}H_2$), 26.0 ($NCH_2\underline{C}H_2$), 49.6 ($NCH_2$), 61.2 (CH), 87.4 (acetylenic carbon), 88.1 (acetylenic carbon), 116.4, 119.1, 121.1, 124.9, 125.6, 127.1, 128.6, 129.4, 129.7, 132.5, 133.8, 157.7 (aromatic carbons). HRMS (ESI) $[M+H]^+$: calcd ($C_{20}H_{21}BrNO$) 370.0807, found 370.0804.

2-(1-(4-Benzylpiperidin-1-yl)-3-phenylprop-2-ynyl)phenol (3h): Yellow viscous liquid; FTIR (Neat): 3365 (w), 3025 (m), 2923 (s), 2849 (s), 2216 (w), 1693 (s), 1605 (s), 1463 (s), 1375 (s), 1276 (s), 1259 (s), 753 (s), 698 (s) cm^{-1}; ^{1}H NMR ($CDCl_3$, 300 MHz): δ 1.27–1.49 (m, 2H), 1.57–1.81 (m, 3H), 2.33–2.40 (m, 1H), 2.54 (d, J = 6.8 Hz, 2H), 2.65–2.74 (m, 2H), 3.01–3.04 (m, 1H), 5.10 (s, 1H, CH), 6.82–6.87 (m, 2H, ArH), 7.12 (d, J = 7 Hz, 2H, ArH), 7.17–7.28 (m, 4H, ArH), 7.33–7.35 (m, 3H, ArH), 7.49–7.55 (m, 3H, ArH); ^{13}C NMR ($CDCl_3$, 75 MHz): δ 31.9 ($NCH_2\underline{C}H_2$), 32.6 ($NCH_2\underline{C}H_2$), 37.7 ($NCH_2CH_2\underline{C}H$), 43.0 (NCH_2), 46.4 ($Ph\underline{C}H_2$), 52.0 (NCH_2), 60.7 (CH), 82.2 (acetylenic carbon), 89.9 (acetylenic carbon), 116.4, 119.1, 121.3, 122.6, 126.0, 128.3, 128.4, 128.6, 129.1, 129.4, 131.9, 140.3, 157.6 (aromatic carbons). HRMS (ESI) $[M+H]^+$: calcd ($C_{27}H_{28}NO$) 382.2171, found 382.2164.

2-(1-(4-Benzylpiperidin-1-yl)-3-*p*-tolylprop-2-ynyl)phenol (3i): Yellow viscous liquid; FTIR (Neat): 3414 (m, br), 2923 (s), 2853 (s), 2219 (w), 1586 (s), 1509 (s), 1463 (s), 1377 (s), 1277 (s), 816 (s), 752 (s), 700 (s) cm^{-1}; ^{1}H NMR ($CDCl_3$, 300 MHz): δ 1.32–1.50 (m, 3H), 1.62–1.83 (m, 3H), 2.34 (s, 3H, CH_3), 2.54–2.56 (m, 2H), 2.66–2.74 (m, 2H), 3.02–3.06 (m, 1H), 5.11 (s, 1H, CH), 6.83–6.88 (m, 2H, ArH), 7.12–7.31 (m, 8H, ArH), 7.40–7.43 (m, 2H, ArH), 7.53–7.57 (m, 1H, ArH); ^{13}C NMR ($CDCl_3$, 75 MHz): δ 21.5 (CH_3), 31.9 ($NCH_2\underline{C}H_2$), 32.6 ($NCH_2\underline{C}H_2$), 37.7 ($NCH_2CH_2\underline{C}H$), 43.0 (NCH_2), 46.4 ($Ph\underline{C}H_2$), 52.0 (NCH_2), 60.7 (CH), 81.5 (acetylenic carbon), 90.0 (acetylenic carbon), 116.3, 119.0, 119.5, 121.5, 126.0, 128.3, 128.5, 129.07, 129.14, 129.4, 131.8, 138.8, 140.3, 157.7 (aromatic carbons). HRMS (ESI) $[M+Na]^+$: calcd ($C_{28}H_{29}NONa$) 418.2147, found 418.2140.

2-(3-Phenyl-1-(pyrrolidin-1-yl)prop-2-ynyl)phenol,[3] (3j): Pale yellow semisolid (Lit.[3] reported as pale yellow oil); FTIR (Neat): 3367 (m), 3056 (s), 2967 (s), 2845 (s), 2268 (w), 1591 (s), 1489 (s), 1466 (s), 1276 (s), 1095 (s), 1258 (s), 754 (s), 691 (s) cm^{-1}; ^{1}H NMR ($CDCl_3$, 300 MHz): δ 1.88–1.90 (m, 4H, 2 x $NCH_2\underline{CH_2}$), 2.79–2.88 (m, 4H, 2 x NCH_2), 5.28 (s, 1H, CH), 6.83–6.88 (m, 2H, ArH), 7.20–7.27 (m, 2H, ArH), 7.35–7.43 (m, 3H, ArH), 7.52–7.55 (m, 3H, ArH); ^{13}C NMR ($CDCl_3$, 75 MHz): δ 23.9 ($NCH_2\underline{C}H_2$), 49.0 (NCH_2), 57.1 (CH), 83.1 (acetylenic carbon), 89.1 (acetylenic carbon), 116.3, 119.0, 122.2, 122.6, 127.9, 128.4, 128.6, 129.4, 131.9, 157.6 (aromatic carbons). HRMS (ESI) $[M+H]^+$: calcd ($C_{19}H_{20}NO$) 278.1545, found 278.1537.

2,4-Dibromo-6-(1-morpholino-3-phenylprop-2-ynyl)phenol (3k): Light yellow solid (mp 63–65 ^{o}C); FTIR (KBr): 3456 (s, br), 3056 (w), 2958 (s), 2849 (s), 2219 (w), 1633 (m), 1597 (m), 1441 (s), 1245 (s), 1117 (s), 754 (s), 707 (s), 690 (s) cm^{-1}; ^{1}H NMR (CDCl$_3$, 300 MHz): δ 1.92 (s, 4H, 2 x OCH$_2$), 2.93 (s, 4H, 2 x NCH$_2$), 4.18 (s, 1H,CH), 6.47–6.53 (m, 3H, ArH), 6.66–6.69 (m, 2H, ArH), 6.74–6.75 (m, 2H, ArH); ^{13}C NMR (CDCl$_3$, 75 MHz): δ 48.2 (NCH$_2$), 59.8 (CH), 65.9 (OCH$_2$), 79.2 (acetylenic carbon), 90.7 (acetylenic carbon), 110.4, 110.6, 120.9, 122.5, 127.8, 128.5, 130.1, 131.2, 134.5, 152.6 (aromatic carbons). HRMS (ESI) [M+Na]$^+$: calcd ($C_{19}H_{17}Br_2NO_2Na$) 471.9524, found 471.9523.

2,4-Dibromo-6-(1-morpholino-3-*p*-tolylprop-2-ynyl)phenol (3l): Light yellow solid (mp 68–70 ^{o}C); FTIR (KBr): 3447 (m, br), 3073 (w), 2952 (s), 2848 (s), 2219 (w), 1636 (w), 1509 (s), 1447 (s), 1247 (s), 1116 (s), 815 (s), 706 (s), 557 (s) cm^{-1}; ^{1}H NMR (CDCl$_3$, 300 MHz): δ 2.39 (s, 3H, CH$_3$), 2.79 (s, 4H, 2 x OCH$_2$), 3.81 (s, 4H, 2 x NCH$_2$), 5.05 (s, 1H, CH), 7.19 (d, J = 7.5 Hz, 2H, ArH), 7.43–7.45 (m, 2H, ArH), 7.62 (s, 2H, ArH); ^{13}C NMR (CDCl$_3$, 75 MHz): δ 21.6 (CH$_3$), 48.7 (NCH$_2$), 60.6 (CH), 66.6 (OCH$_2$), 79.2 (acetylenic carbon), 91.6 (acetylenic carbon), 111.1, 111.3, 118.5, 123.4, 129.3, 130.9, 131.9, 135.2, 139.5, 153.3 (aromatic carbons). HRMS (ESI) [M+Na]$^+$: calcd ($C_{20}H_{19}Br_2NO_2Na$) 485.9681, found 485.9679.

2-Methoxy-6-(1-morpholino-3-phenylprop-2-ynyl)phenol,[4] (3m): Yellow solid (mp 130–132 ^{o}C); FTIR (KBr): 3438 (m, br), 2935 (m), 2846 (s), 1584 (m), 1457 (s), 1243 (s), 1116 (s), 1072 (s), 988 (s), 855 (s), 753 (s) cm^{-1}. ^{1}H NMR (CDCl$_3$, 300 MHz) δ 2.71 (s, 4H, 2 x OCH$_2$), 3.76 (s, 4H, 2 x NCH$_2$), 3.80 (s, 3H, OCH$_3$), 6.72–6.82 (m, 2H, ArH), 7.10–7.12 (m, 1H, ArH), 7.23–7.29 (m, 3H, ArH), 7.42–7.44 (m, 2H, ArH), 10.97 (s, br, 1H, OH).^{13}C NMR (CDCl$_3$, 75 MHz): δ 48.9 (NCH$_2$), 56.0 (OCH$_3$), 60.6 (CH), 66.8 (OCH$_2$), 81.7 (acetylenic carbon), 90.1 (acetylenic carbon), 111.9, 118.9, 120.6, 120.8, 122.2, 128.4, 128.7, 131.9, 146.4, 148.0 (aromatic carbons).

2-Methoxy-6-(1-morpholino-3-*p*-tolylprop-2-ynyl)phenol (3n): White solid (mp 172–174 ^{o}C); FTIR (KBr): 3402 (w), 2921 (s), 2837 (s), 2211 (m), 1584 (s), 1466 (s), 1238 (s), 1112 (s), 1075 (s), 987 (s), 820 (s), 749 (s) cm^{-1}. ^{1}H NMR (CDCl$_3$, 300 MHz): δ 2.39 (s, 3H, CH$_3$), 2.80 (s, 4H, 2 x OCH$_2$), 3.81 (s, 4H, 2 x NCH$_2$), 3.90 (s, 3H, OCH$_3$), 5.11 (s, 1H, CH), 6.82–6.91 (m, 2H, ArH), 7.16–7.23 (m, 3H, ArH), 7.45 (d, J = 7.8 Hz, 2H, ArH). ^{13}C NMR (CDCl$_3$, 75 MHz): δ 21.5 (CH$_3$), 48.9 (NCH$_2$), 55.9 (OCH$_3$), 60.6, 66.8 (OCH$_2$), 80.9 (acetylenic carbon), 90.2 (acetylenic carbon), 111.8, 118.8, 119.1, 120.7, 120.9, 129.1, 131.7, 138.9, 146.3, 148.0 (aromatic carbons).

2-(3-(2-Bromophenyl)-1-morpholinoprop-2-ynyl)-6-methoxyphenol (3o): Orange sticky solid; FTIR (KBr): 3413 (m), 2924 (s), 2851 (s), 2230 (w) 1586 (m), 1467 (s), 1240 (w), 1116 (s), 1082 (s), 991 (s), 747 (s), 567 (s) cm^{-1}. ^{1}H NMR (CDCl$_3$, 300 MHz): δ 2.85 (s, 4H, 2 x OCH$_2$), 3.81 (s, 4H, 2 x NCH$_2$), 3.89 (s, 3H, OCH$_3$), 5.17 (s, 1H, CH), 6.81–6.91 (m, 2H, ArH), 7.19–7.33 (m, 3H, ArH), 7.56–7.64 (m, 2H, ArH), 10.98 (s, br, 1H, OH). ^{13}C NMR (CDCl$_3$, 75 MHz): δ 49.1 (NCH$_2$), 56.0 (OCH$_3$), 60.7 (CH), 66.9 (OCH$_2$), 86.6 (acetylenic

carbon), 88.4 (acetylenic carbon), 111.9, 118.9, 120.5, 120.8, 124.5, 125.5, 127.1, 129.9, 132.5, 133.7, 146.3, 148.0 (aromatic carbons).

2-(3-(4-Bromophenyl)-1-morpholinoprop-2-ynyl)-6-methoxyphenol (3p): Pale yellow solid (mp 156–158 ^{o}C); FTIR (KBr): 3428 (m), 2931 (s), 2847 (s), 2207 (w), 1594 (m), 1459 (s), 1240 (s), 1115 (s), 1070 (s), 982 (s), 822 (s), 744 (s) cm^{-1}. ^{1}H NMR ($CDCl_3$, 300 MHz): δ 2.78 (s, 4H, 2 x OCH_2), 3.80 (s, 4H, 2 x NCH_2), 3.89 (s, 3H, OCH_3), 5.08 (s, 1H, CH), 6.80–6.91 (m, 2H, ArH), 7.12–7.15 (m, 1H, ArH), 7.37–7.41 (m, 2H, ArH),7.47–7.50 (m, 2H, ArH), 10.92 (s, br, 1H, OH). ^{13}C NMR ($CDCl_3$, 75 MHz): δ 49.0 (NCH_2), 56.0 (OCH_3), 60.6 (CH), 66.8 (OCH_2), 83.06 (acetylenic carbon), 89.0 (acetylenic carbon), 111.9, 118.9, 120.5, 120.6, 121.1, 123.0, 131.7, 133.3, 146.3, 148.1 (aromatic carbons). HRMS (ESI) $[M+Na]^+$: calcd ($C_{20}H_{20}BrNO_3Na$) 424.0524, found 424.0525.

2-(1-(4-Benzylpiperidin-1-yl)-3-phenylprop-2-ynyl)-6-methoxyphenol (3q): Pale yellow solid (mp 118–119 ^{o}C); FTIR (KBr): 3428 (m), 2930 (s), 2827 (s), 1587 (m), 1497 (s), 1375 (s), 1324 (s), 1242 (s), 1073 (s), 832 (s), 749 (s), 697 (s), 590 (s) cm^{-1}. ^{1}H NMR ($CDCl_3$, 300 MHz) δ 1.28–1.41 (m, 2H), 1.51–1.62 (m, 2H), 1.72–1.76 (m, 1H), 2.29–2.36 (m, 1H), 2.46 (d, J = 6.78 Hz, 2H), 2.59–2.71 (m, 2H), 2.96–3.00 (m, 1H), 3.83 (s, 3H, OCH_3), 5.07 (s, 1H, CH), 6.71–6.82 (m, 2H), 7.05–7.07 (m, 2H), 7.10–7.14 (m, 2H, ArH), 7.18–7.22 (m, 2H, ArH), 7.26–7.30 (m, 3H, ArH), 7.44–7.47 (m, 2H, ArH). ^{13}C NMR ($CDCl_3$, 75 MHz): δ 31.7 ($NCH_2\underline{C}H_2$), 32.6 ($NCH_2\underline{C}H_2$), 37.6 ($NCH_2CH_2\underline{C}H$), 42.9 (NCH_2), 46.4 ($PhCH_2$), 52.0 (NCH_2), 56.0 (OCH_3), 60.6 (CH), 82.2 (acetylenic carbon), 89.6 (acetylenic carbon), 111.6, 118.4, 120.3, 121.5, 122.5, 125.9, 128.2, 128.4, 128.6, 129.0, 131.8, 140.3, 147.1, 148.1 (aromatic carbons). HRMS (ESI) $[M+Na]^+$: calcd ($C_{28}H_{29}NO_2Na$) 434.2096, found 434.2092.

2-(1-(4-Benzylpiperidin-1-yl)-3-*p*-tolylprop-2-ynyl)-6-methoxyphenol (3r): White solid (mp 155–156 ^{o}C); FTIR (KBr): 3407 (m, br), 2929 (s), 2837 (s), 1594 (m), 1458 (s), 1237 (s), 1075 (s), 989 (s), 820 (s), 731 (s) cm^{-1}. ^{1}H NMR ($CDCl_3$, 300 MHz) δ 1.27–1.38 (m, 2H), 1.52–1.61 (m, 2H), 1.71–1.76 (m, 1H), 2.30–2.35 (m, 4H), 2.46 (d, J = 6.9 Hz, 2H), 2.60–2.70 (m, 2H), 2.95–2.99 (m, 1H), 3.82 (s, 3H, OCH_3), 5.05 (S, 1H, CH), 6.70–6.81 (m, 2H), 7.04–7.13 (m, 6H), 7.18–7.22 (m, 2H, ArH), 7.34 (d, J = 8.1 Hz, 2H).^{13}C NMR ($CDCl_3$, 75 MHz): δ 21.5 (CH_3), 31.7 ($NCH_2\underline{C}H_2$), 32.5 ($NCH_2\underline{C}H_2$), 37.6 ($NCH_2CH_2\underline{C}H$), 42.9 (NCH_2), 46.3 ($PhCH_2$), 51.9 (NCH_2), 56.0 (OCH_3), 60.6 (CH), 81.5 (acetylenic carbon), 89.7 (acetylenic carbon), 111.6, 118.3, 119.4, 120.4, 121.6, 125.9, 128.2, 129.0, 129.1, 131.7, 138.7, 140.3, 147.1, 148.0 (aromatic carbons).

2-Methoxy-6-(3-phenyl-1-(piperidin-1-yl)prop-2-ynyl)phenol (3s): White solid (mp 132–134 ^{o}C); FTIR (KBr): 3417 (w, br), 2930 (s), 2837 (s), 1584 (m), 1458 (s), 1239 (s), 1074 (s), 985 (s), 742 (s), 587 (s) cm^{-1}. ^{1}H NMR ($CDCl_3$, 300 MHz) δ 1.51 (s, 2H, $N(CH_2)_2\underline{CH_2}$), 1.66–1.69 (m, 4H, 2 x $NCH_2\underline{CH_2}$), 2.73 (s, 4H, 2 x NCH_2), 3.89 (s, 3H, OCH_3), 5.10 (s, 1H, CH), 6.77–6.89 (m, 2H, ArH), 7.17–7.20 (m, 1H, ArH), 7.34–7.37 (m, 3H, ArH), 7.53–7.56 (m, 2H, ArH). ^{13}C NMR ($CDCl_3$, 75 MHz): δ 24.0 ($N(CH_2)_2\underline{C}H_2$), 25.9 ($NCH_2\underline{C}H_2$), 49.7 ($NCH_2$), 56.0 ($OCH_3$), 61.0 (CH), 82.4 (acetylenic carbon), 89.5 (acetylenic carbon), 111.6, 118.3, 120.4, 121.5, 122.6, 128.4, 128.5, 131.8, 147.1, 148.0 (aromatic carbons).

2-Methoxy-6-(1-(piperidin-1-yl)-3-*p*-tolylprop-2-ynyl)phenol (3t): Yellowish white solid (mp 152–154 ^oC); FTIR (KBr): 3423 (w), 2916 (s), 2832 (s), 1594 (m), 1466 (s), 1240 (s), 1078 (s), 987 (s), 822 (s), 741 (s) cm^{-1}. ^1H NMR ($CDCl_3$, 300 MHz) δ 1.43 (s, 2H, $N(CH_2)_2\underline{CH_2}$), 1.61 (s, 4H, 2 x $NCH_2\underline{CH_2}$), 2.29 (s, 4H, 2 x NCH_2), 2.65 (s, 3H, CH_3), 3.81 (s, 3H, OCH_3), 5.01 (s, 1H, CH), 6.67–6.80 (m, 2H, ArH), 6.98–7.29 (m, 3H, ArH), 7.35 (d, 2H, J = 8.1 Hz, ArH), 7.35 (d, 2H, J = 8.1 Hz ArH). ^{13}C NMR ($CDCl_3$, 75 MHz): δ 21.5 (CH_3), 24.0 ($N(CH_2)_2\underline{C}H_2$), 25.9 ($NCH_2\underline{C}H_2$), 49.5 ($NCH_2$), 55.9 ($OCH_3$), 61.0 (CH), 81.6 (acetylenic carbon), 89.6 (acetylenic carbon), 111.5, 118.3, 119.5, 120.4, 121.6, 129.1, 131.7, 138.7, 147.1, 148.0 (aromatic carbons). HRMS (ESI) $[M+Na]^+$: calcd ($C_{22}H_{25}NO_2Na$) 358.1783, found 358.1779.

IV. **References**

1. Namitharan, K.; Pitchumani, K. *Eur. J. Org. Chem.* **2010,** 411–415. doi: 10.1002/ejoc.200901084
2. Borah, B. J.; Borah, S. J.; Saikia, K.; Dutta D. K. *Catal. Sci. Technol.* **2014,** *4*, 4001–4009. doi:10.1039/C4CY00666F
3. Trose, M.; Dell'Acqua, M.; Pedrazzini, T.; Pirovano, V.; Gallo, E.; Rossi, E.; Caselli, A.; Abbiati, G. *J. Org. Chem.* **2014,** *79*, 7311–7320. doi:10.1021/jo500981r
4. Mirabedini, M.; Motamedi, E.; Kassaee, M. Z. *Chin. Chem. Lett.* **2015,** *26*, 1085–1090. doi:10.1016/j.cclet.2015.05.021

V. Scanned spectra (^{1}H and ^{13}C NMR) of propargylamine derivatives (3a–t)

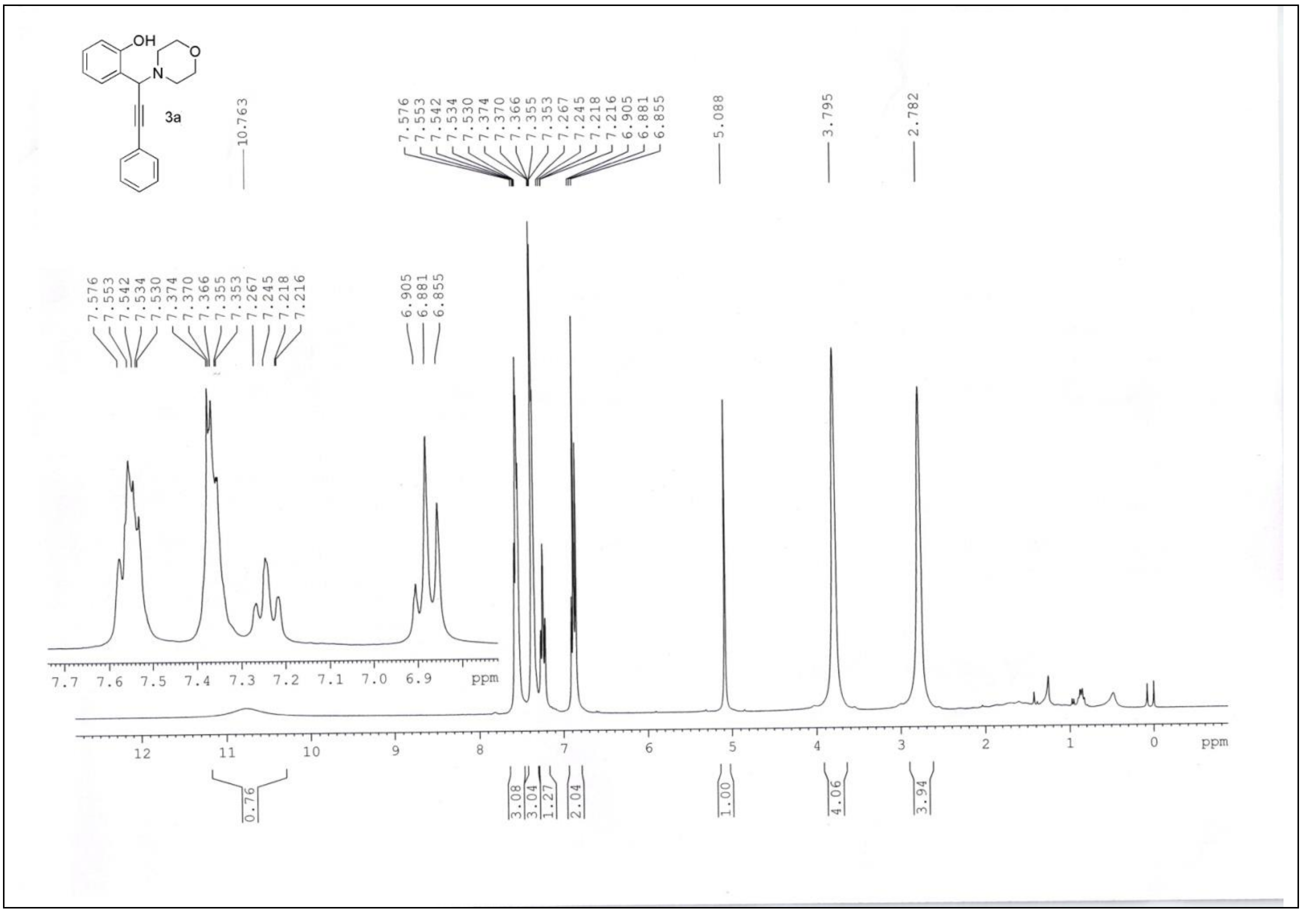

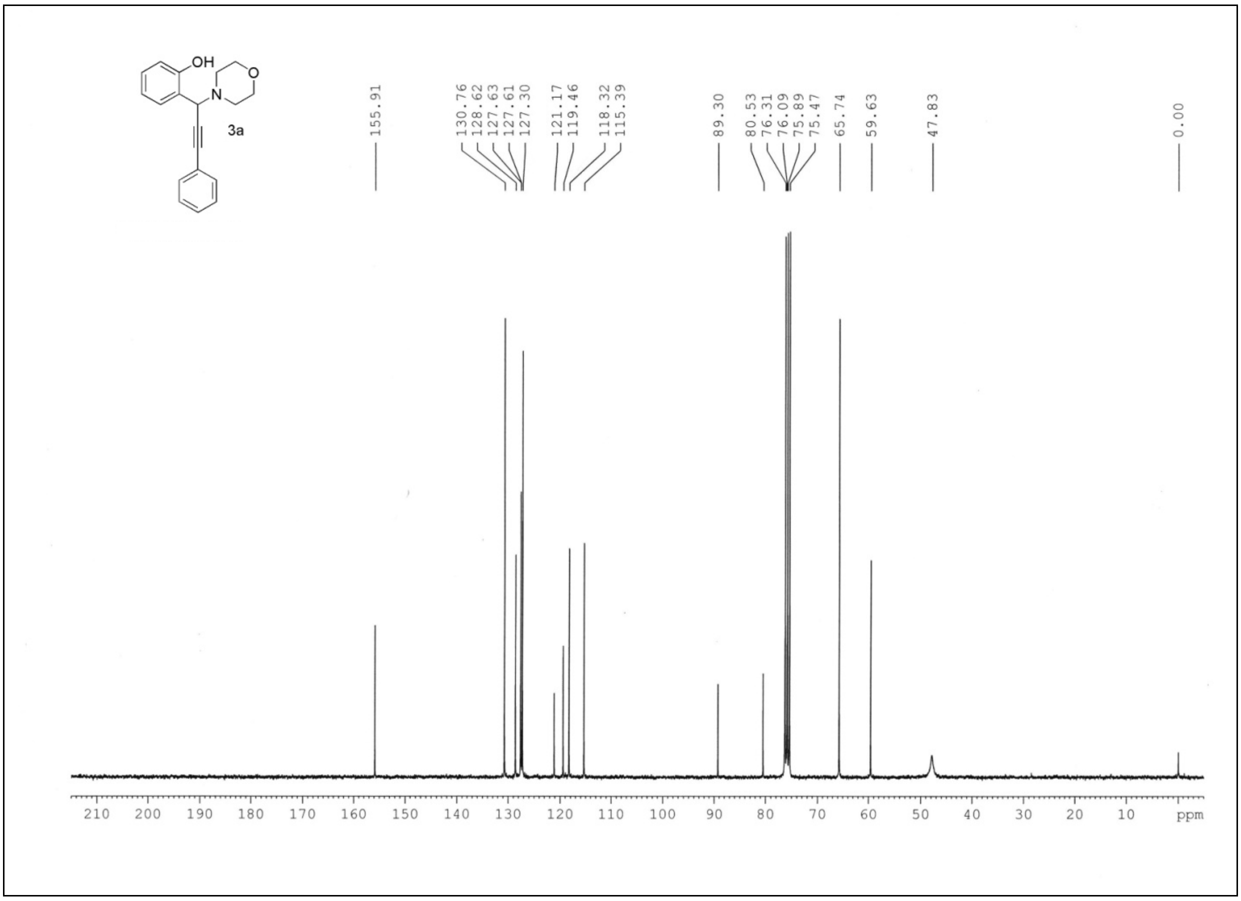
OH
N
O
3a
155.91
130.76
128.62
127.63
127.61
127.30
121.17
119.46
118.32
115.39
89.30
80.53
76.31
76.09
75.89
75.47
65.74
59.63
47.83
0.00
210 200 190 180 170 160 150 140 130 120 110 100 90 80 70 60 50 40 30 20 10 ppm

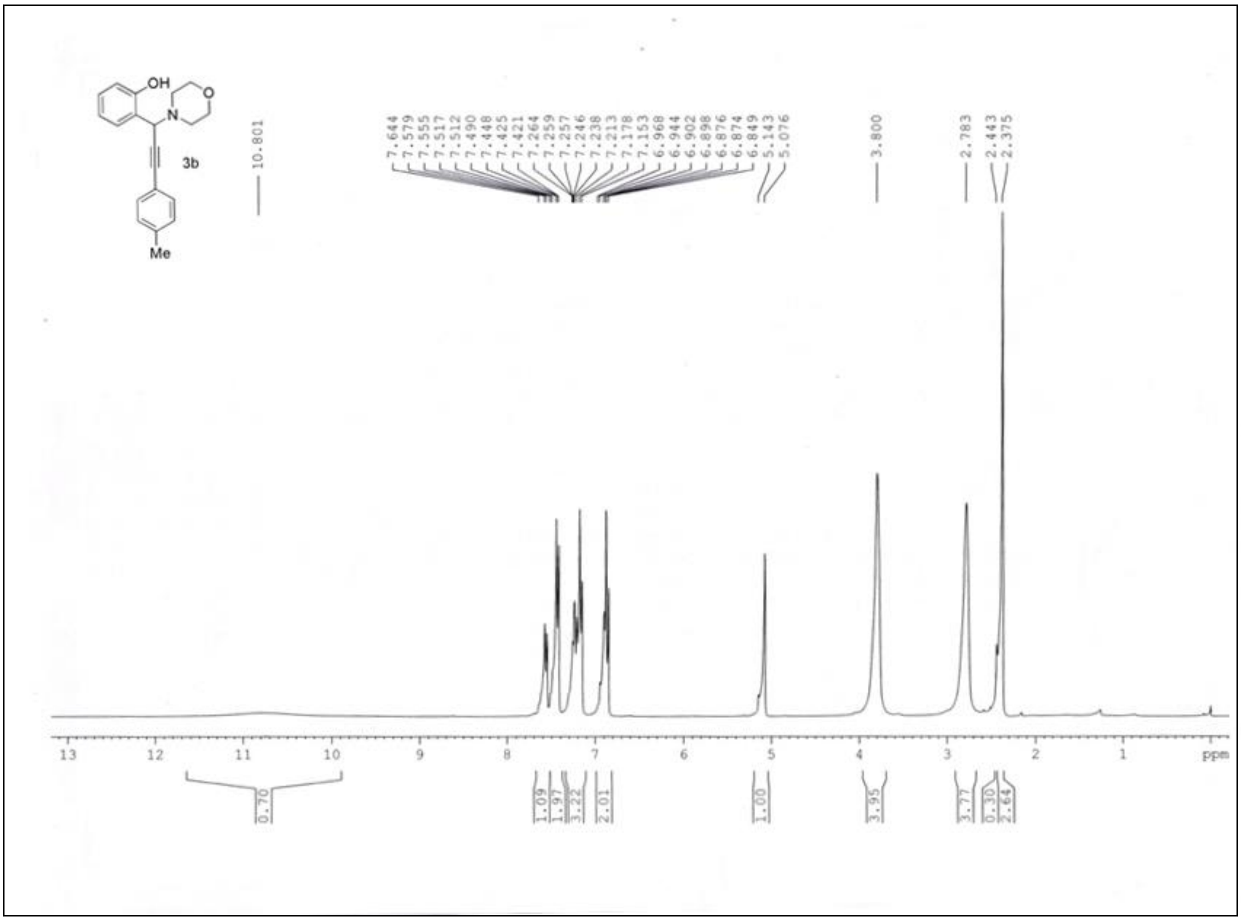
3b
OH
N
O
Me
10.801
7.644
7.579
7.555
7.517
7.512
7.490
7.448
7.425
7.421
7.264
7.259
7.257
7.246
7.238
7.213
7.178
7.153
6.968
6.944
6.902
6.898
6.876
6.874
6.849
5.143
5.076
3.800
2.783
2.443
2.375
13 12 11 10 9 8 7 6 5 4 3 2 1 ppm
0.70
1.09
1.97
3.22
2.01
1.00
3.95
3.77
0.30
2.64

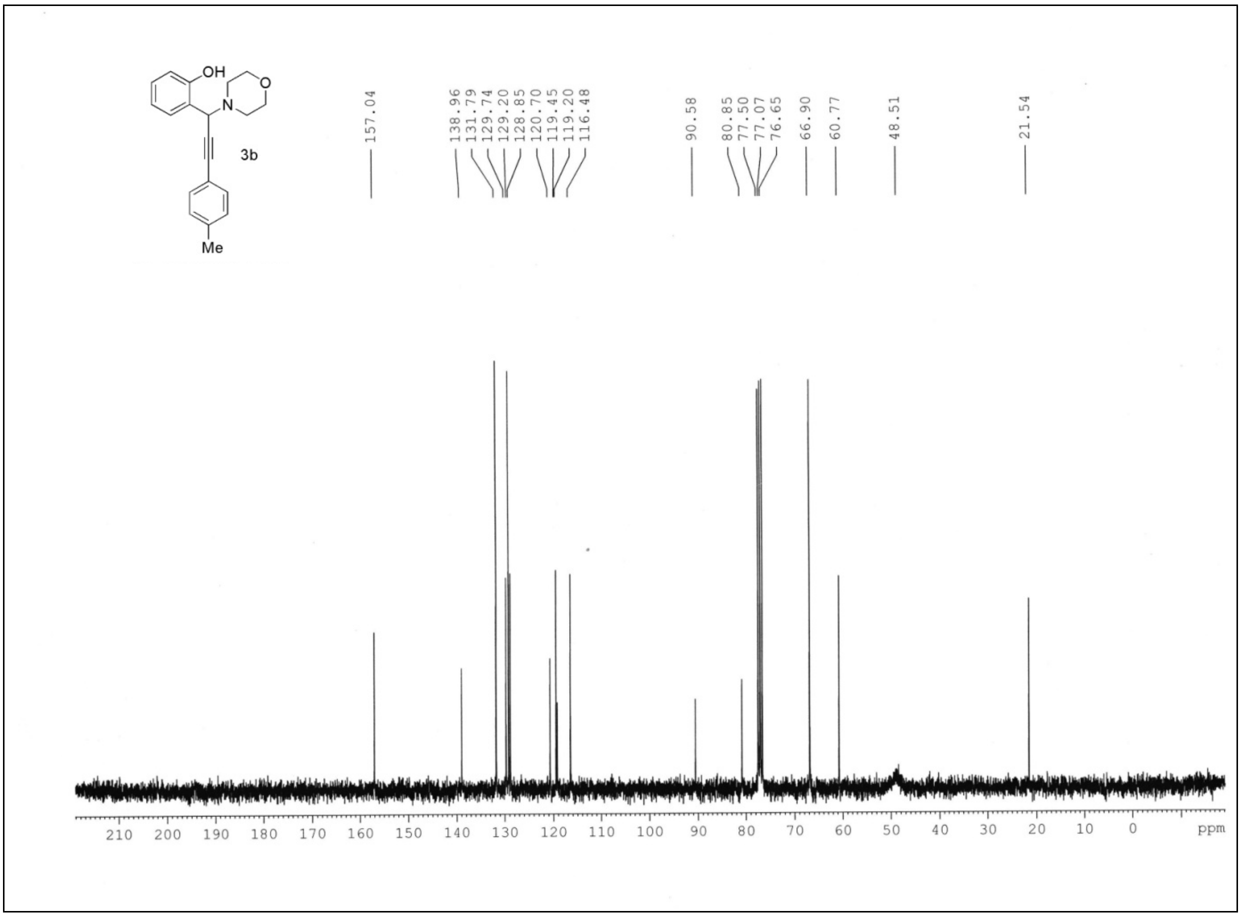
OH
N
O
3b
Me
157.04
138.96
131.79
129.74
129.20
128.85
120.70
119.45
119.20
116.48
90.58
80.85
77.50
77.07
76.65
66.90
60.77
48.51
21.54
210 200 190 180 170 160 150 140 130 120 110 100 90 80 70 60 50 40 30 20 10 0 ppm

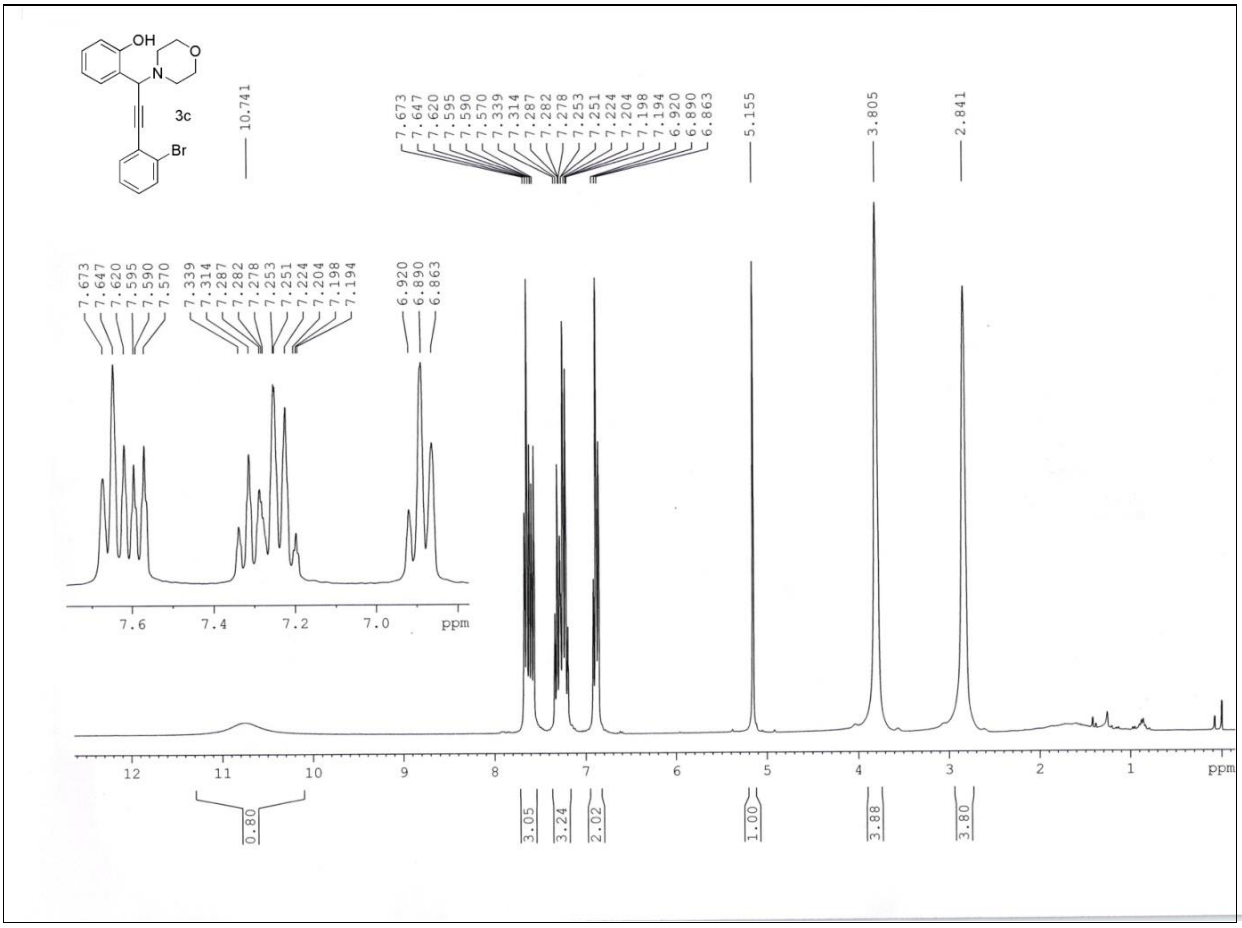

OH
N
O
3c
Br
10.741
7.673
7.647
7.620
7.595
7.590
7.570
7.339
7.314
7.287
7.282
7.278
7.253
7.251
7.224
7.204
7.198
7.194
6.920
6.890
6.863
5.155
3.805
2.841
7.6
7.4
7.2
7.0
ppm
12
11
10
9
8
7
6
5
4
3
2
1
ppm
0.80
3.05
3.24
2.02
1.00
3.88
3.80

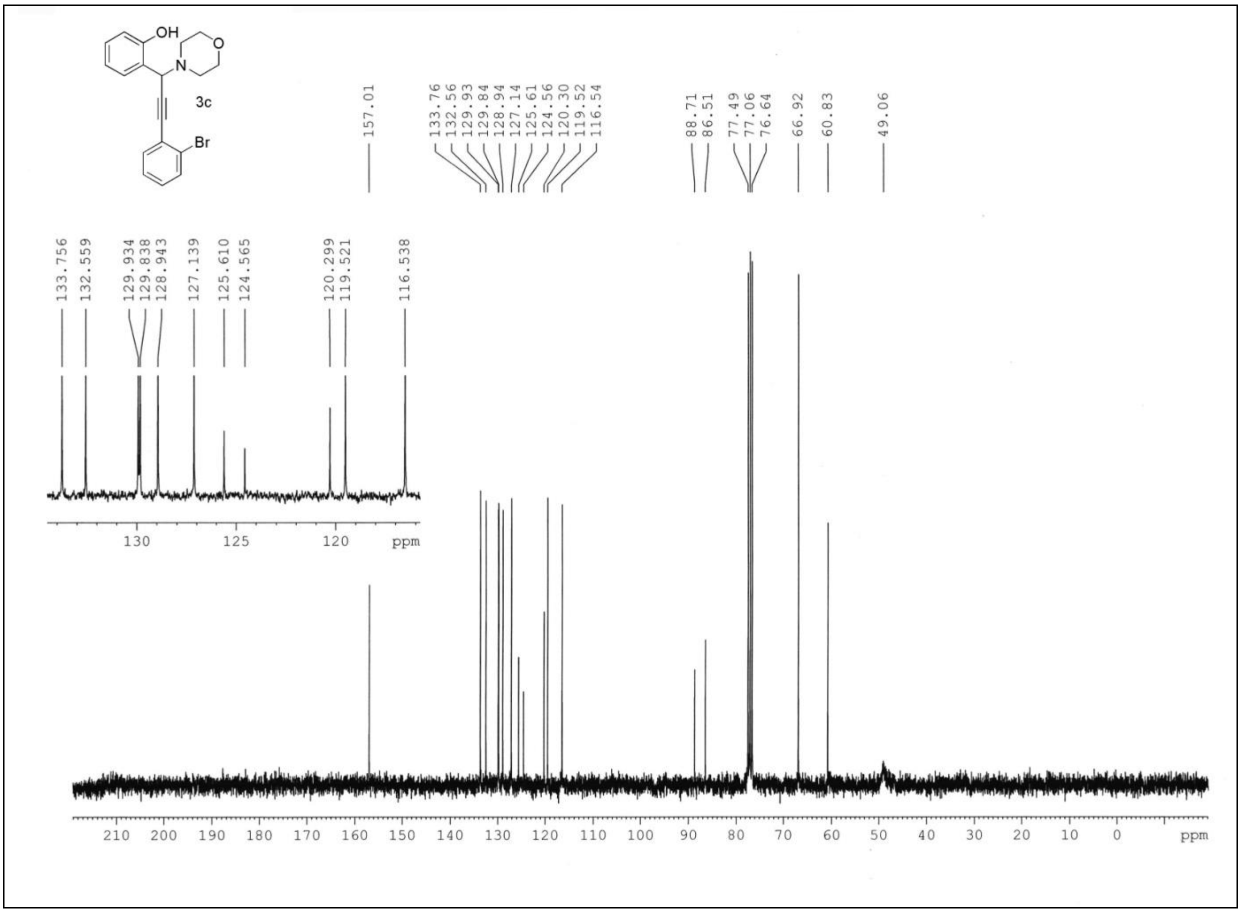
OH
N
O
3c
Br
157.01
133.76
132.56
129.93
129.84
128.94
127.14
125.61
124.56
120.30
119.52
116.54
88.71
86.51
77.49
77.06
76.64
66.92
60.83
49.06
133.756
132.559
129.934
129.838
128.943
127.139
125.610
124.565
120.299
119.521
116.538
130 125 120 ppm
210 200 190 180 170 160 150 140 130 120 110 100 90 80 70 60 50 40 30 20 10 0 ppm

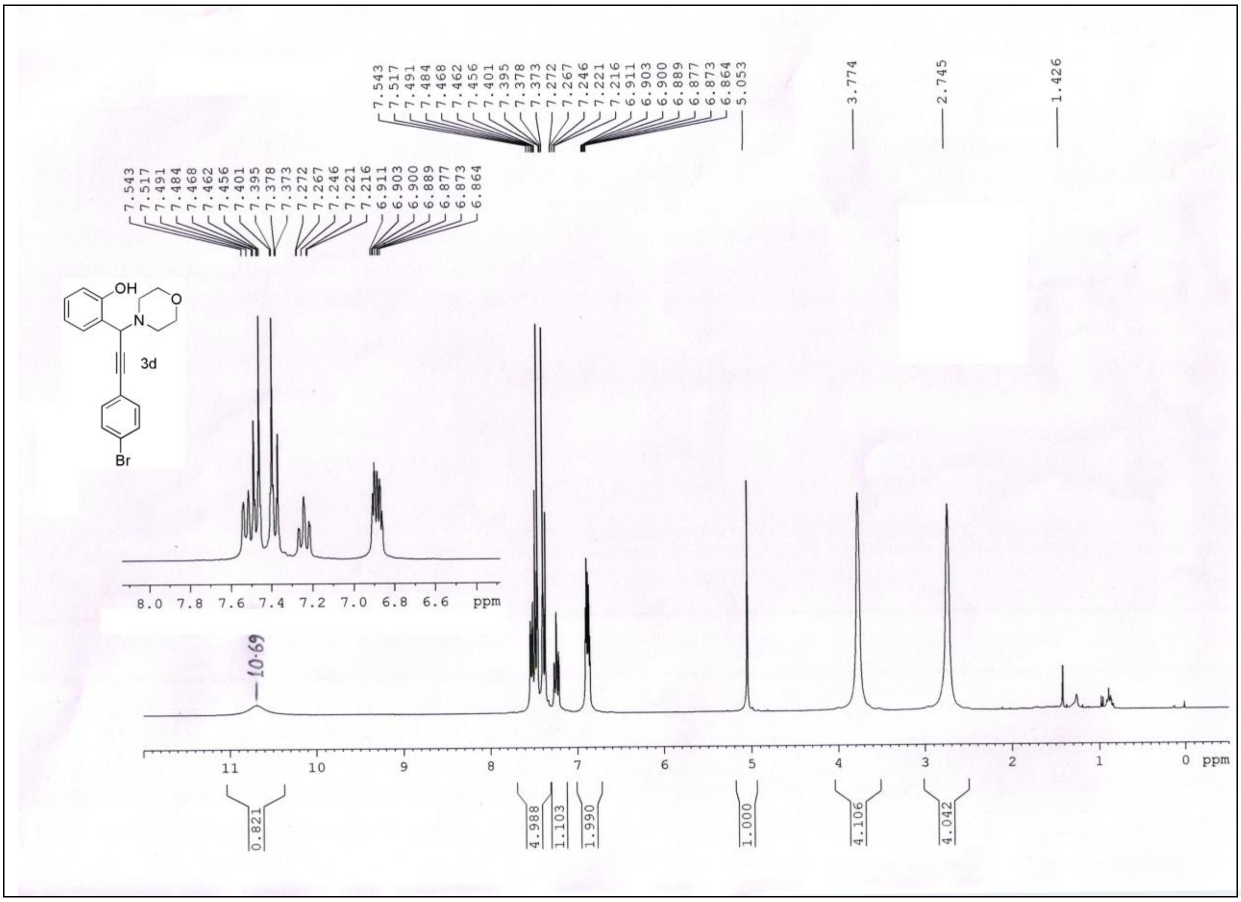

3d
OH
N
O
Br
7.543
7.517
7.491
7.484
7.468
7.462
7.456
7.401
7.395
7.378
7.373
7.272
7.267
7.246
7.221
7.216
6.911
6.903
6.900
6.889
6.877
6.873
6.864
5.053
3.774
2.745
1.426
10.69
0.821
4.988
1.103
1.990
1.000
4.106
4.042
ppm

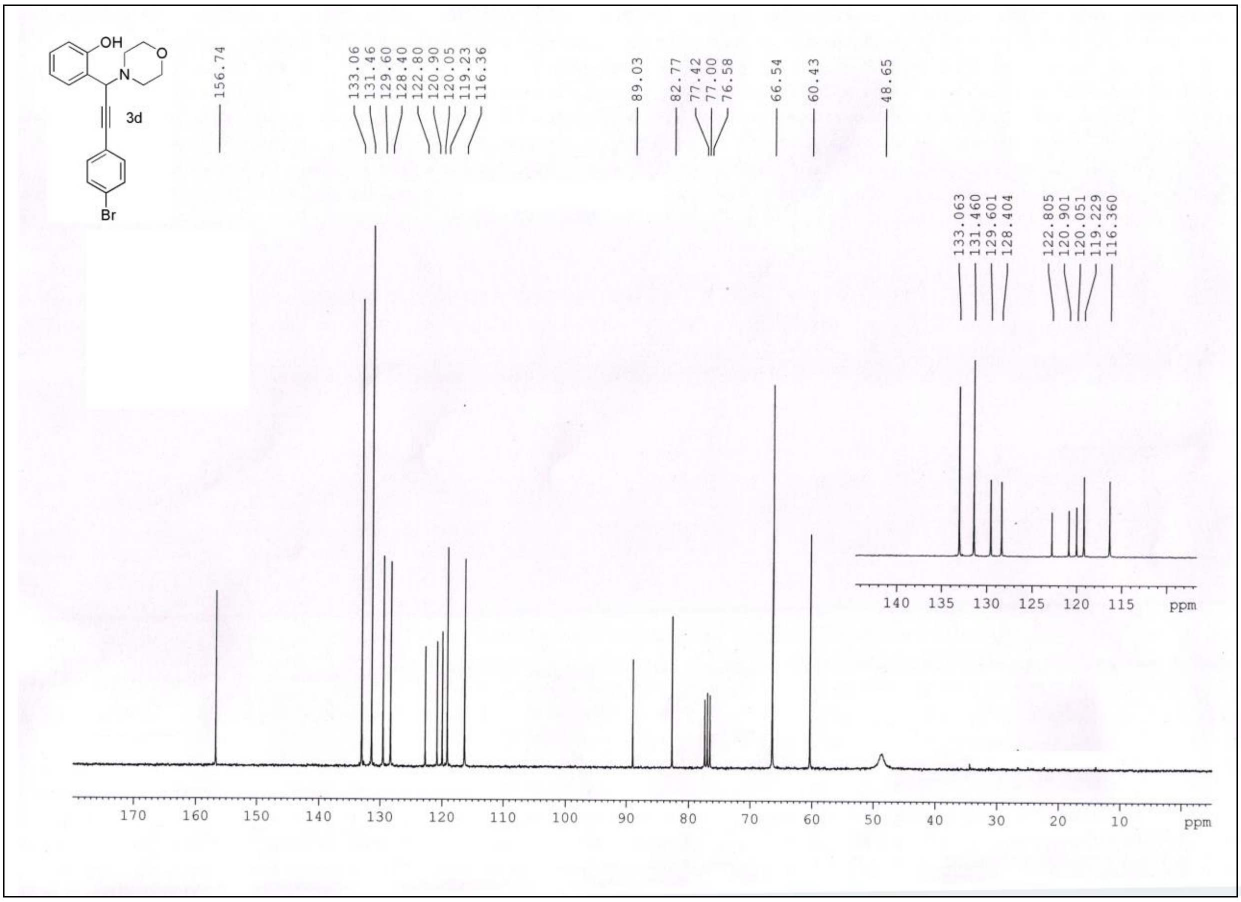
OH
N
O
3d
Br
156.74
133.06
131.46
129.60
128.40
122.80
120.90
120.05
119.23
116.36
89.03
82.77
77.42
77.00
76.58
66.54
60.43
48.65
133.063
131.460
129.601
128.404
122.805
120.901
120.051
119.229
116.360
140 135 130 125 120 115 ppm
170 160 150 140 130 120 110 100 90 80 70 60 50 40 30 20 10 ppm

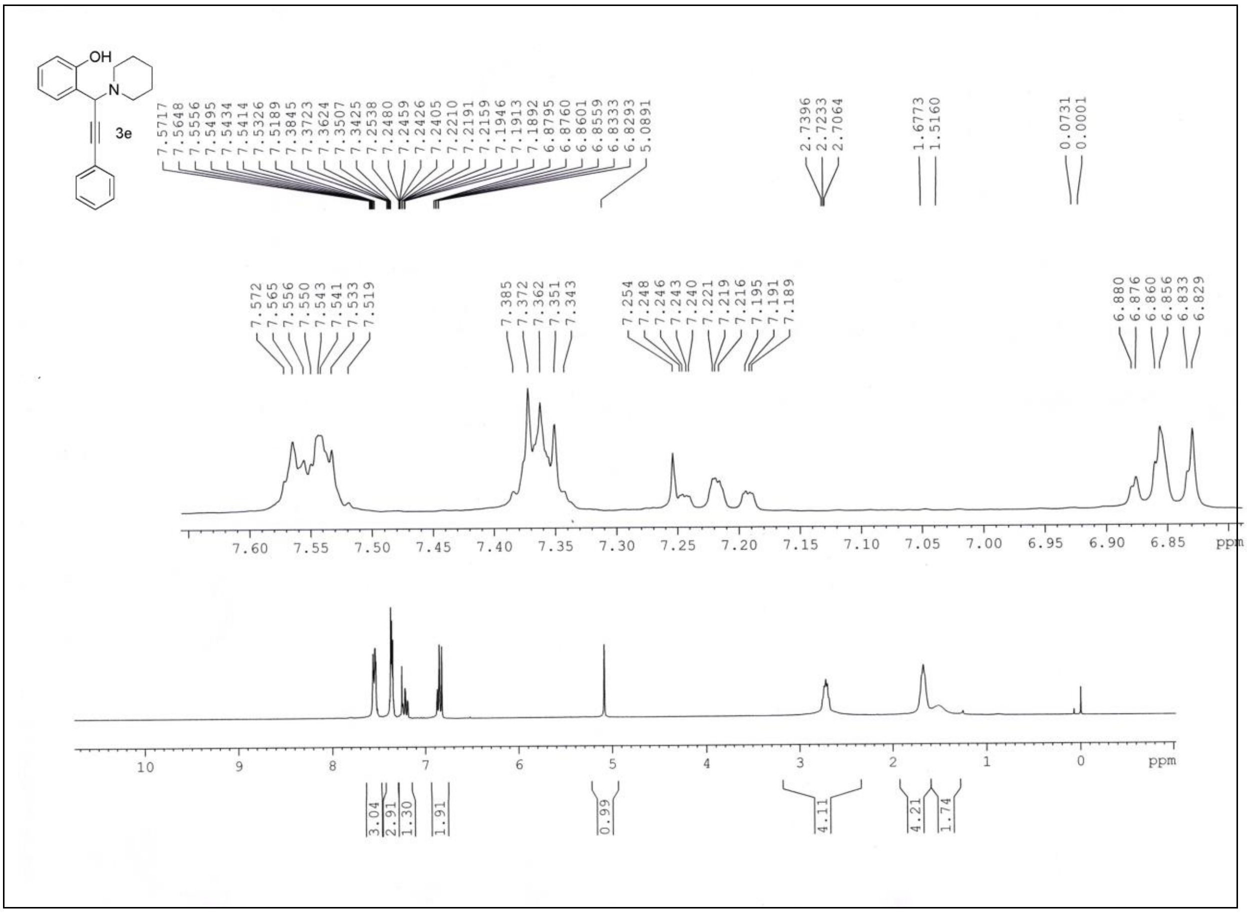
OH
N
3e
7.5717
7.5648
7.5556
7.5495
7.5434
7.5414
7.5326
7.5189
7.3845
7.3723
7.3624
7.3507
7.3425
7.2538
7.2480
7.2459
7.2426
7.2405
7.2210
7.2191
7.2159
7.1946
7.1913
7.1892
6.8795
6.8760
6.8601
6.8559
6.8333
6.8293
5.0891
2.7396
2.7233
2.7064
1.6773
1.5160
0.0731
0.0001
ppm
3.04
2.91
1.30
1.91
0.99
4.11
4.21
1.74

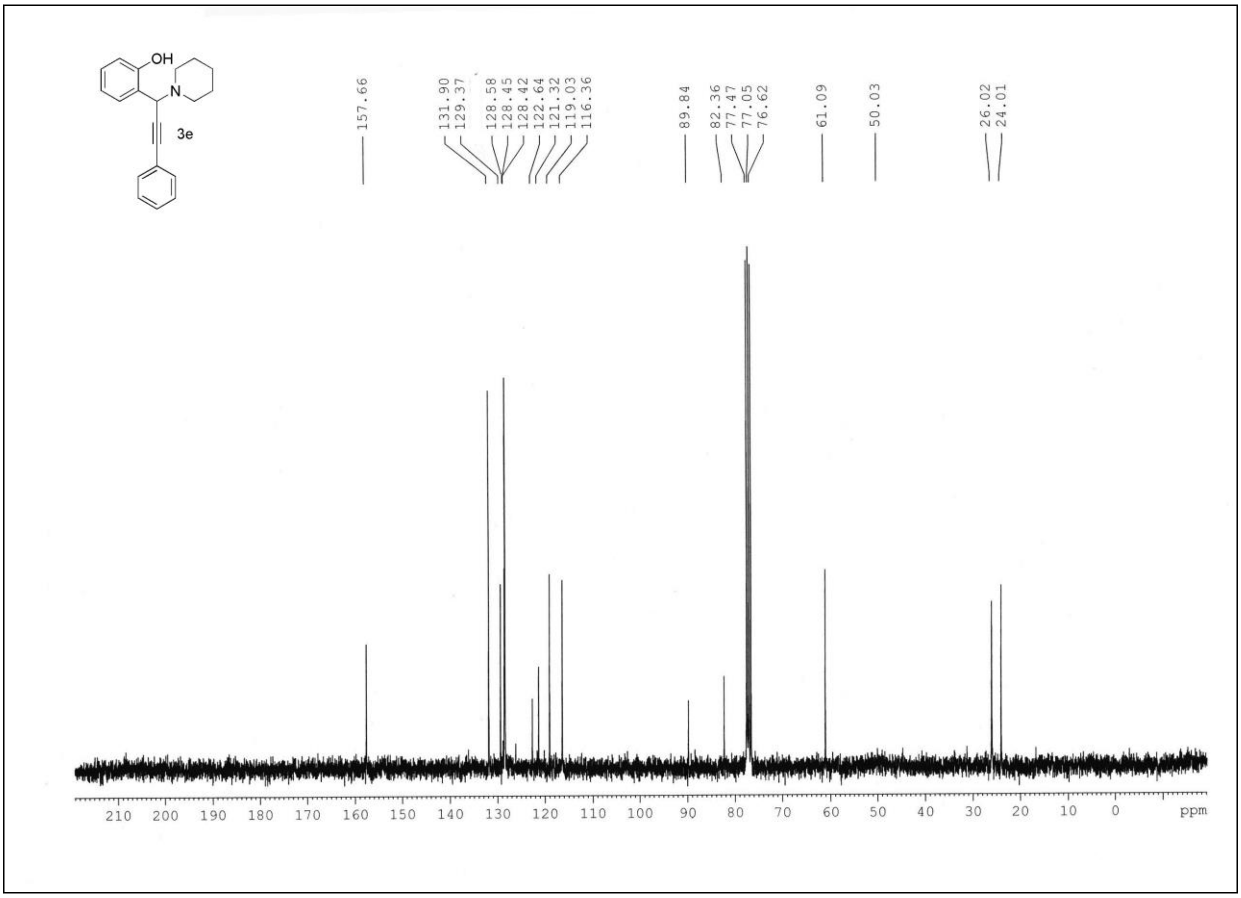
OH
N
3e
157.66
131.90
129.37
128.58
128.45
128.42
122.64
121.32
119.03
116.36
89.84
82.36
77.47
77.05
76.62
61.09
50.03
26.02
24.01
210 200 190 180 170 160 150 140 130 120 110 100 90 80 70 60 50 40 30 20 10 0 ppm

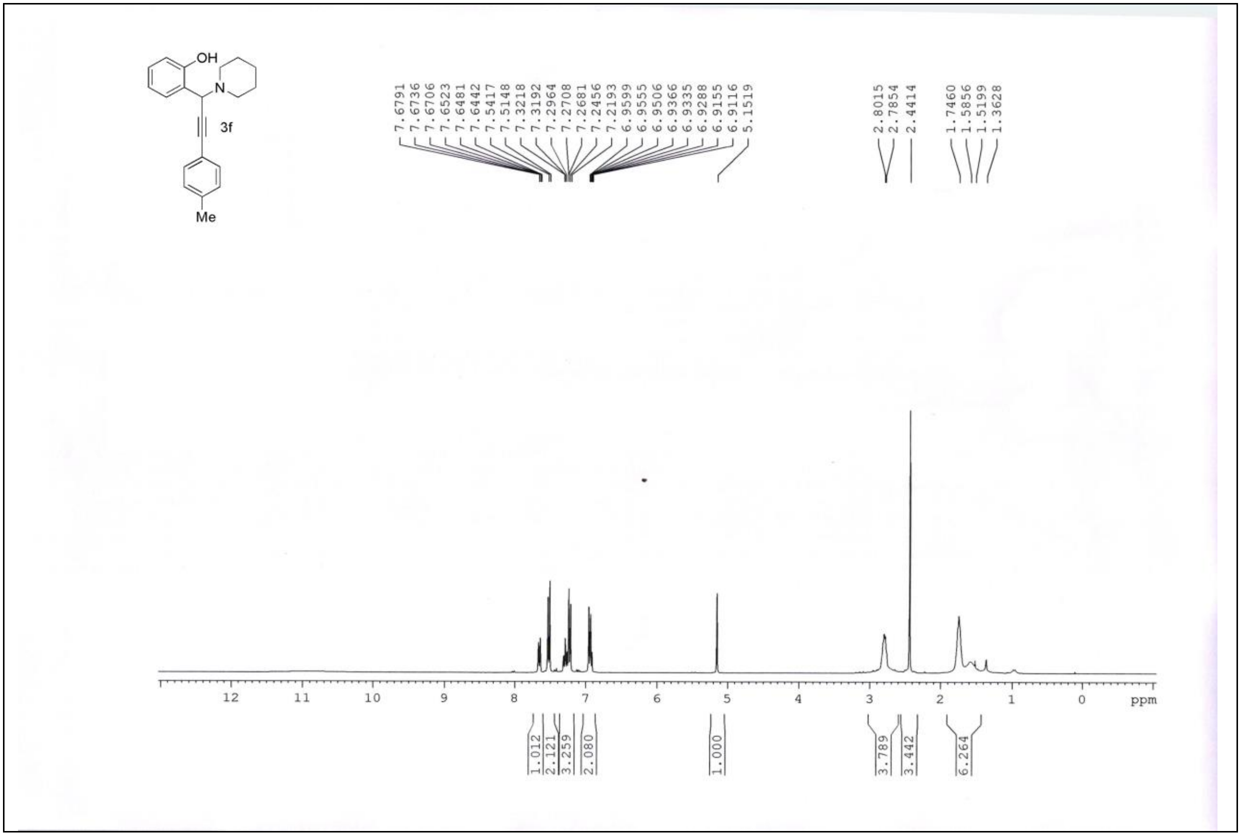
OH
N
3f
Me
7.6791
7.6736
7.6706
7.6523
7.6481
7.6442
7.5417
7.5148
7.3218
7.3192
7.2964
7.2708
7.2681
7.2456
7.2193
6.9599
6.9555
6.9506
6.9366
6.9335
6.9288
6.9155
6.9116
5.1519
2.8015
2.7854
2.4414
1.7460
1.5856
1.5199
1.3628
12
11
10
9
8
7
6
5
4
3
2
1
0
ppm
1.012
2.121
3.259
2.080
1.000
3.789
3.442
6.264

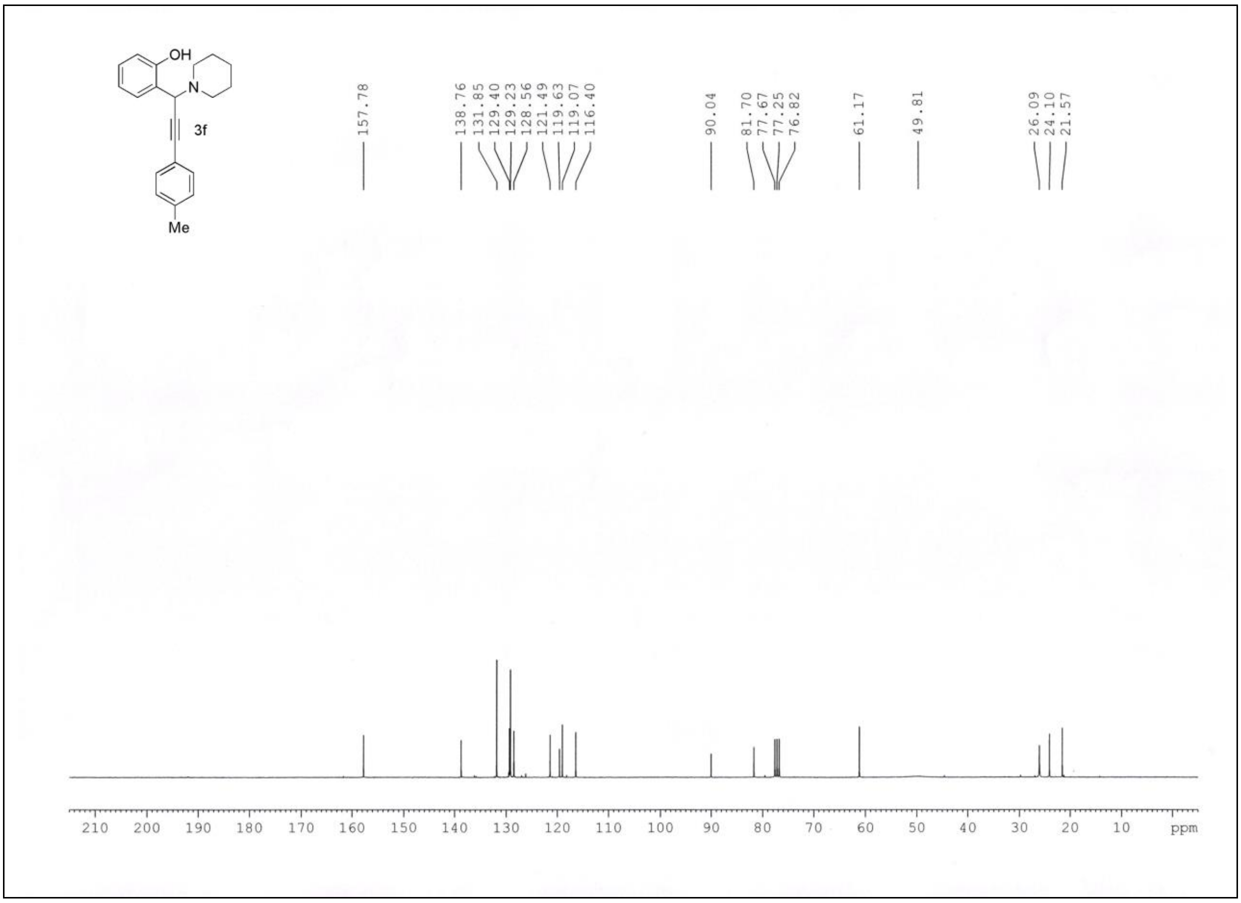
OH
N
3f
Me
157.78
138.76
131.85
129.40
129.23
128.56
121.49
119.63
119.07
116.40
90.04
81.70
77.67
77.25
76.82
61.17
49.81
26.09
24.10
21.57
210 200 190 180 170 160 150 140 130 120 110 100 90 80 70 60 50 40 30 20 10 ppm

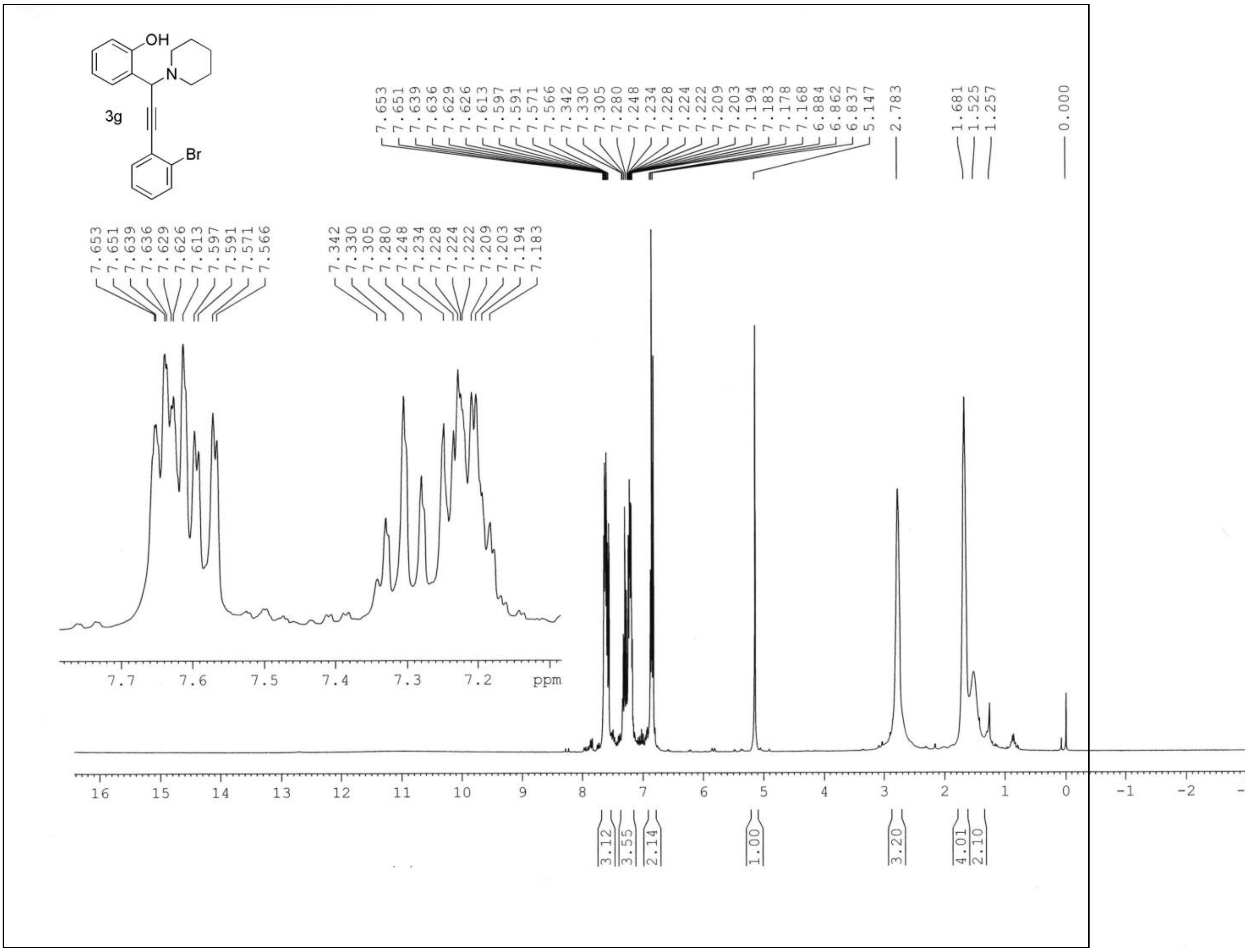
OH
N
3g
Br
7.653
7.651
7.639
7.636
7.629
7.626
7.613
7.597
7.591
7.571
7.566
7.342
7.330
7.305
7.280
7.248
7.234
7.228
7.224
7.222
7.209
7.203
7.194
7.183
7.178
7.168
6.884
6.862
6.837
5.147
2.783
1.681
1.525
1.257
0.000
7.7 7.6 7.5 7.4 7.3 7.2 ppm
16 15 14 13 12 11 10 9 8 7 6 5 4 3 2 1 0 -1 -2
3.12
3.55
2.14
1.00
3.20
4.01
2.10

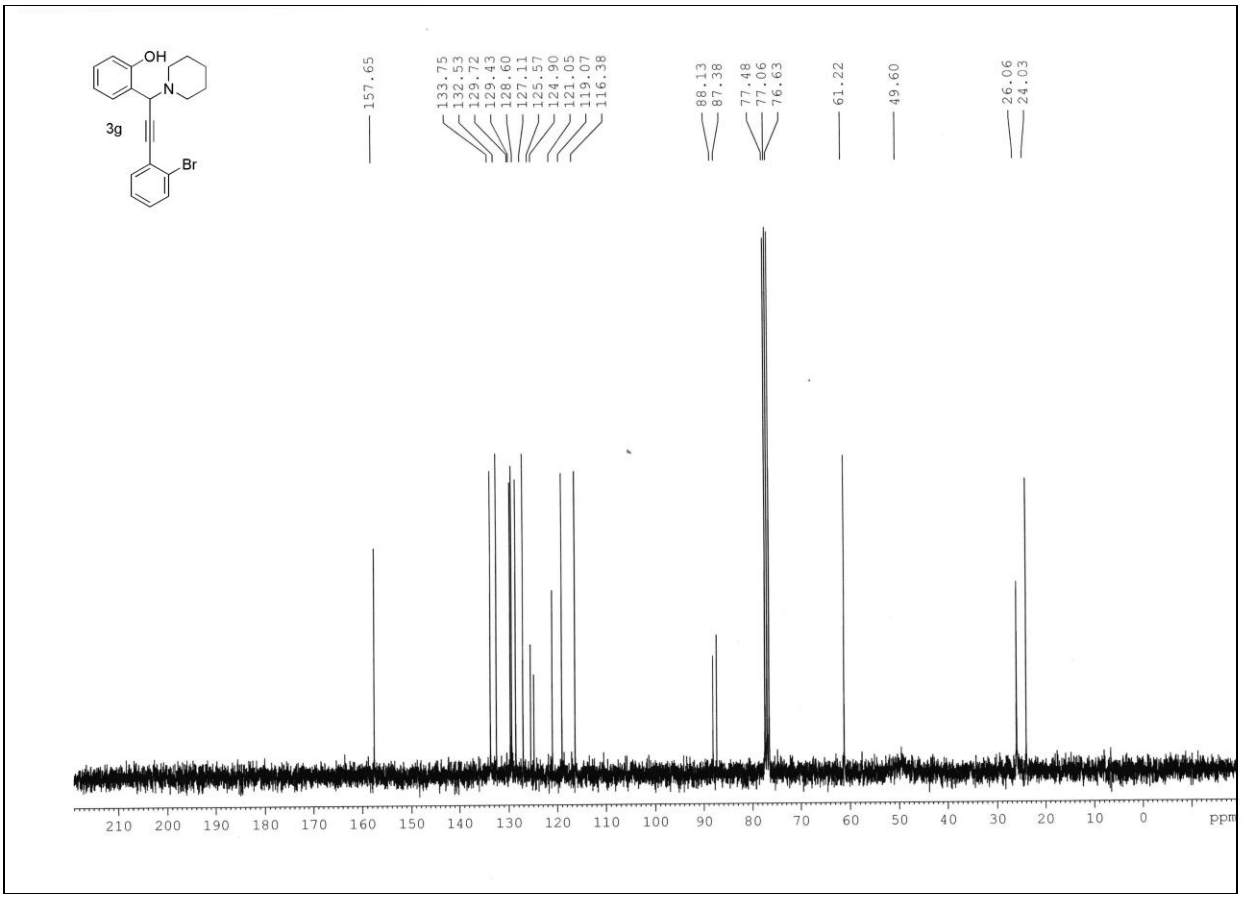
OH
N
3g
Br
157.65
133.75
132.53
129.72
129.43
128.60
127.11
125.57
124.90
121.05
119.07
116.38
88.13
87.38
77.48
77.06
76.63
61.22
49.60
26.06
24.03
210 200 190 180 170 160 150 140 130 120 110 100 90 80 70 60 50 40 30 20 10 0 ppm

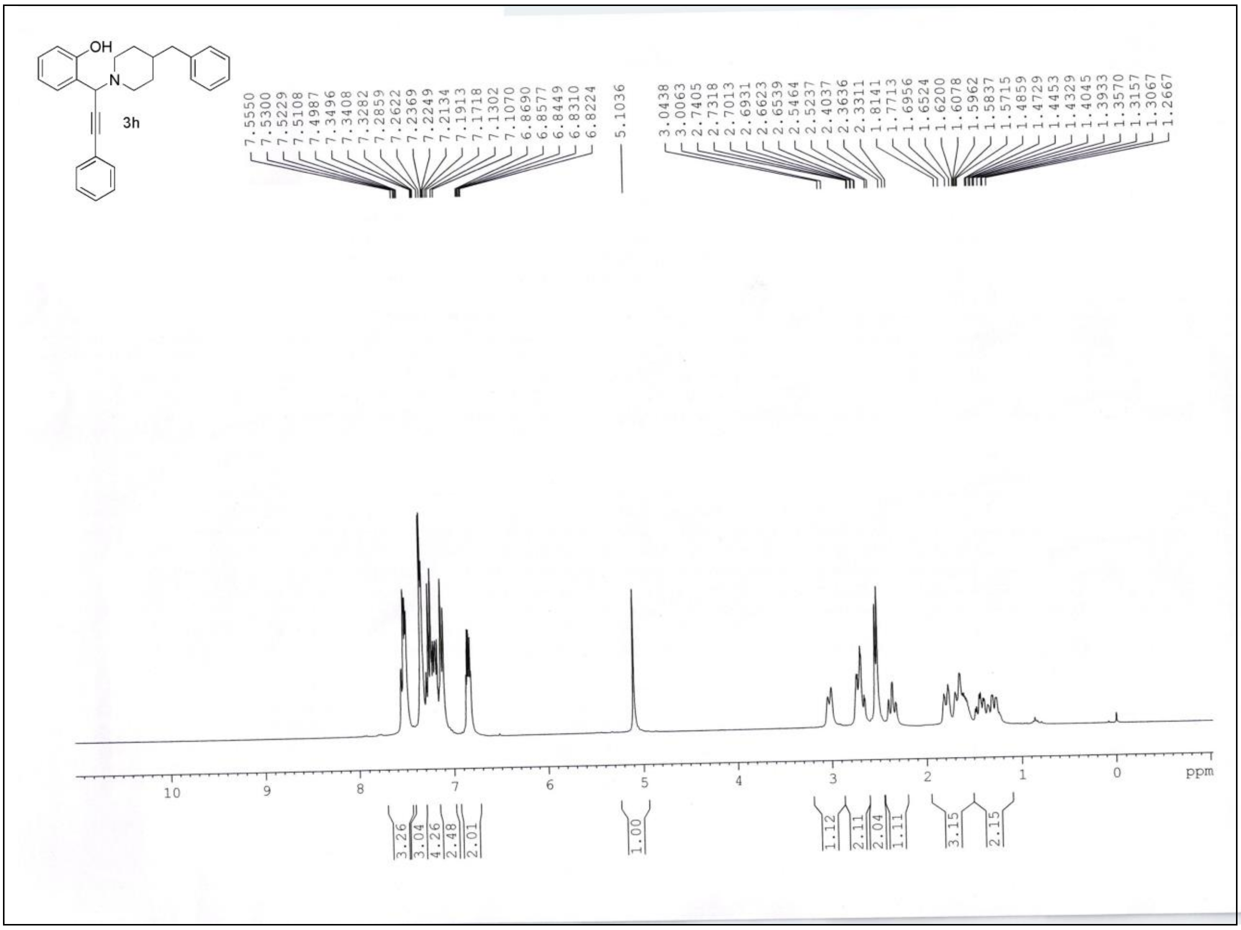
OH
N
3h
ppm

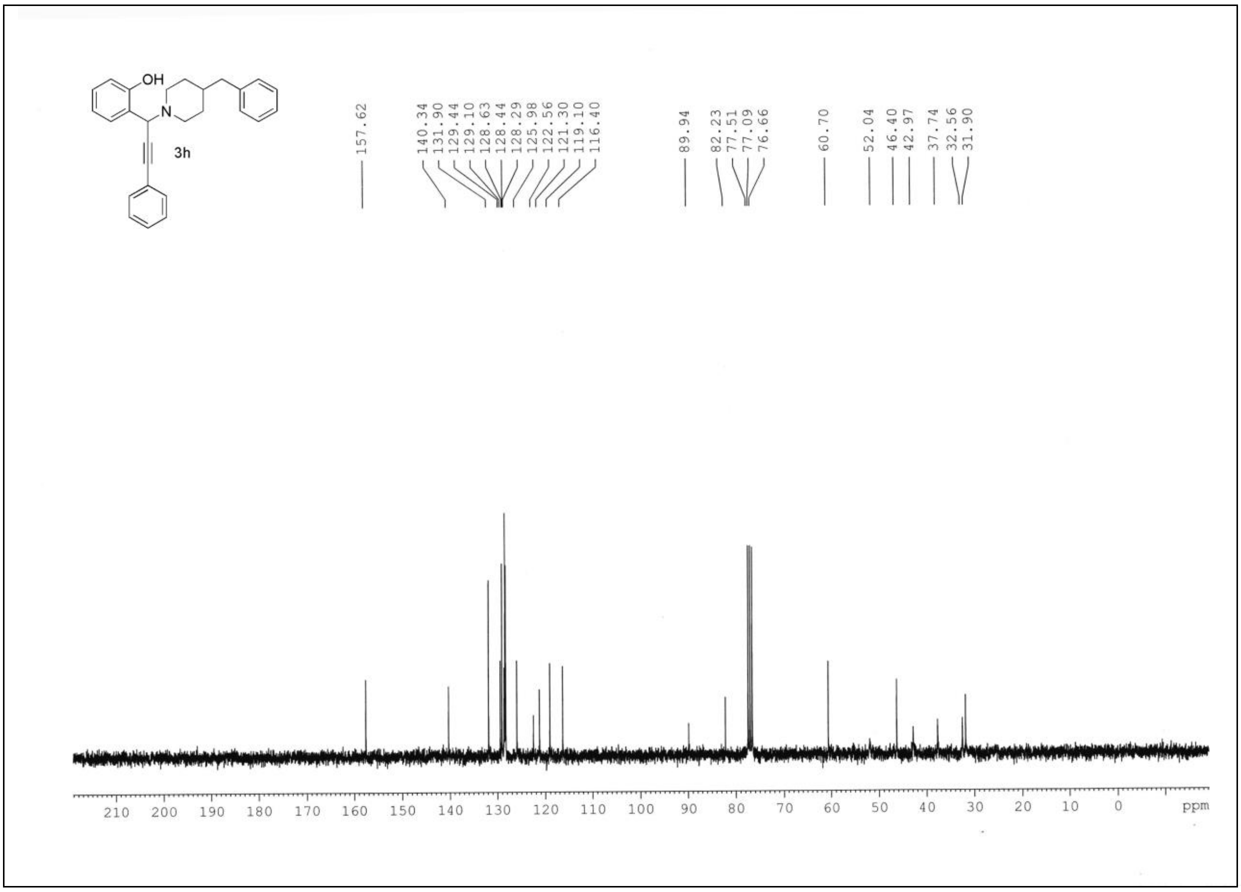
OH
N
3h
157.62
140.34
131.90
129.44
129.10
128.63
128.44
128.29
125.98
122.56
121.30
119.10
116.40
89.94
82.23
77.51
77.09
76.66
60.70
52.04
46.40
42.97
37.74
32.56
31.90
210 200 190 180 170 160 150 140 130 120 110 100 90 80 70 60 50 40 30 20 10 0
ppm

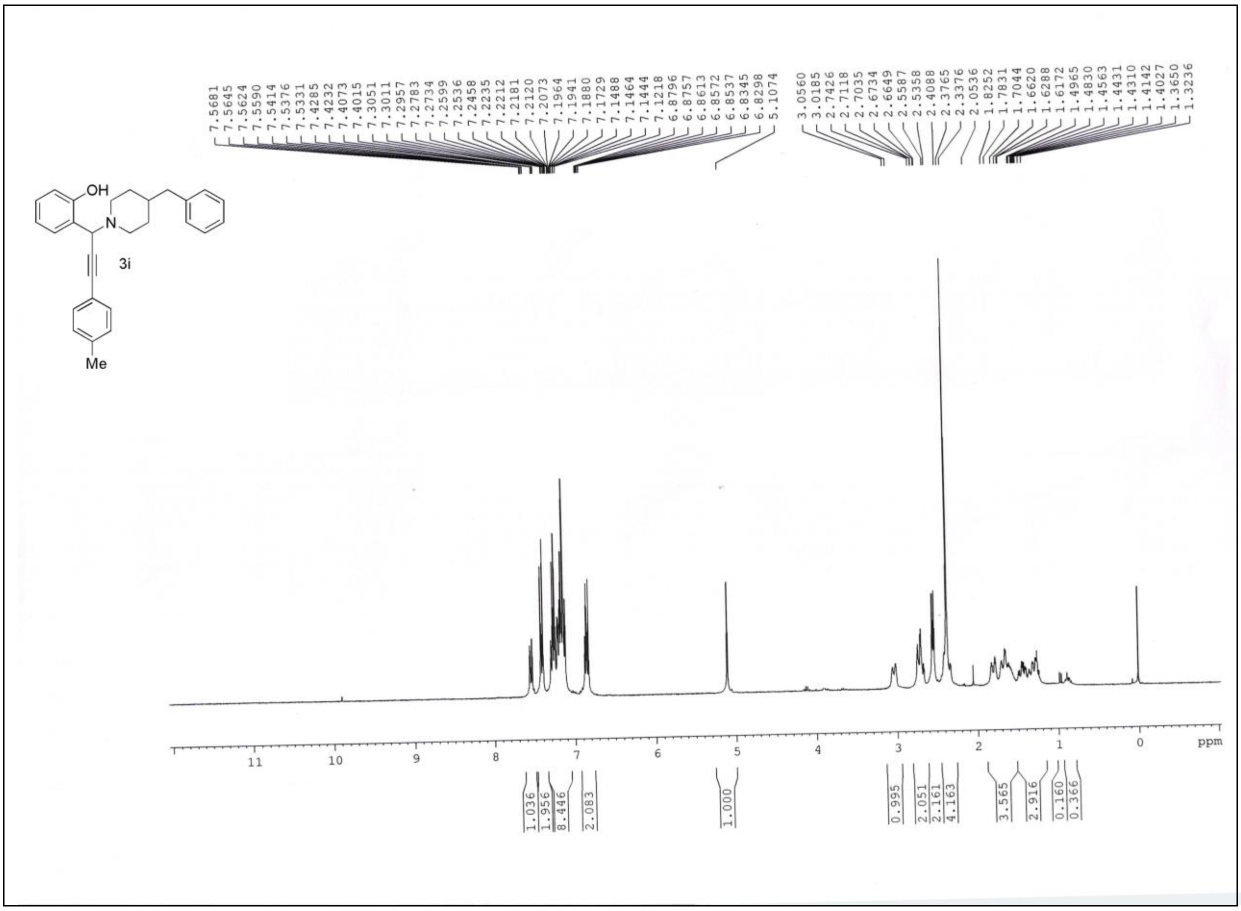
OH
N
3i
Me
ppm

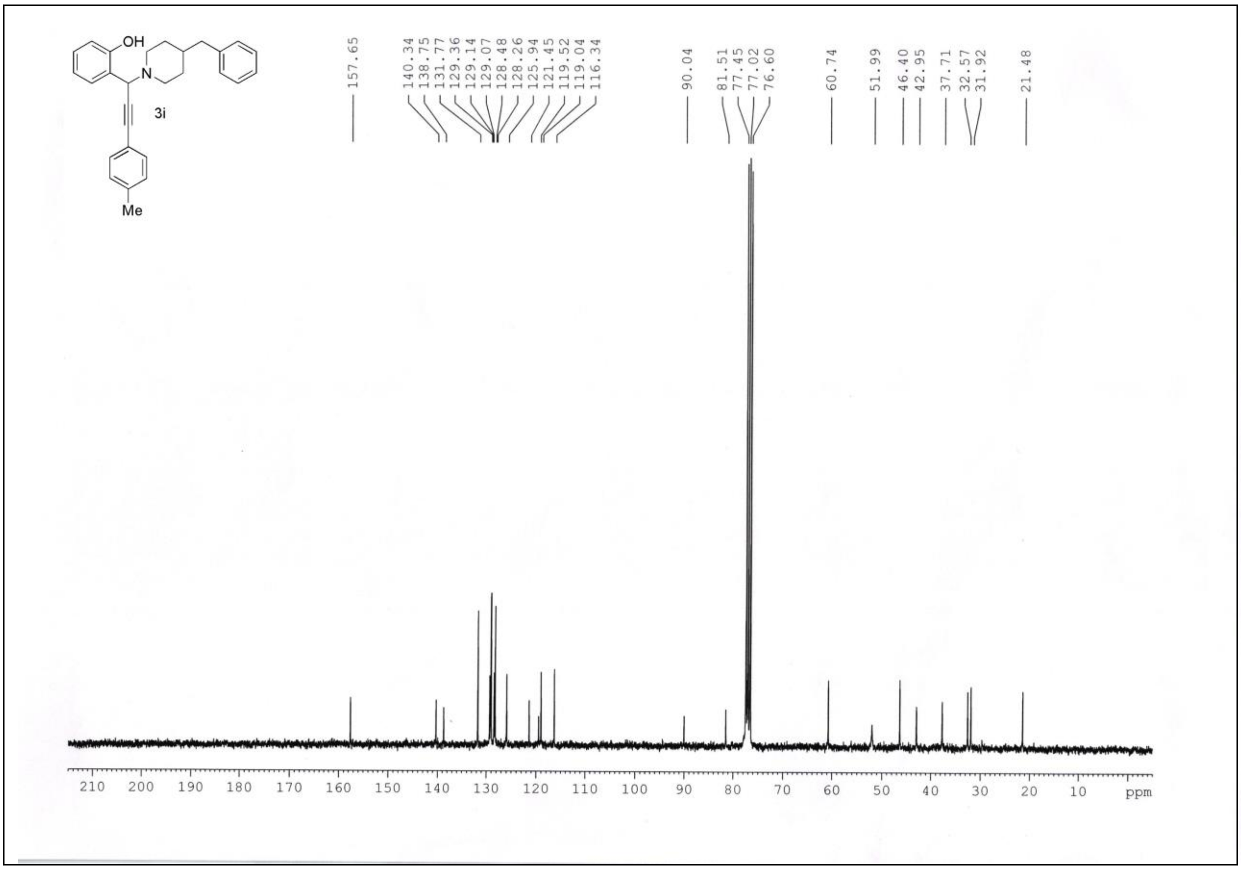
OH
N
3i
Me
157.65
140.34
138.75
131.77
129.36
129.14
129.07
128.48
128.26
125.94
121.45
119.52
119.04
116.34
90.04
81.51
77.45
77.02
76.60
60.74
51.99
46.40
42.95
37.71
32.57
31.92
21.48
210 200 190 180 170 160 150 140 130 120 110 100 90 80 70 60 50 40 30 20 10 ppm

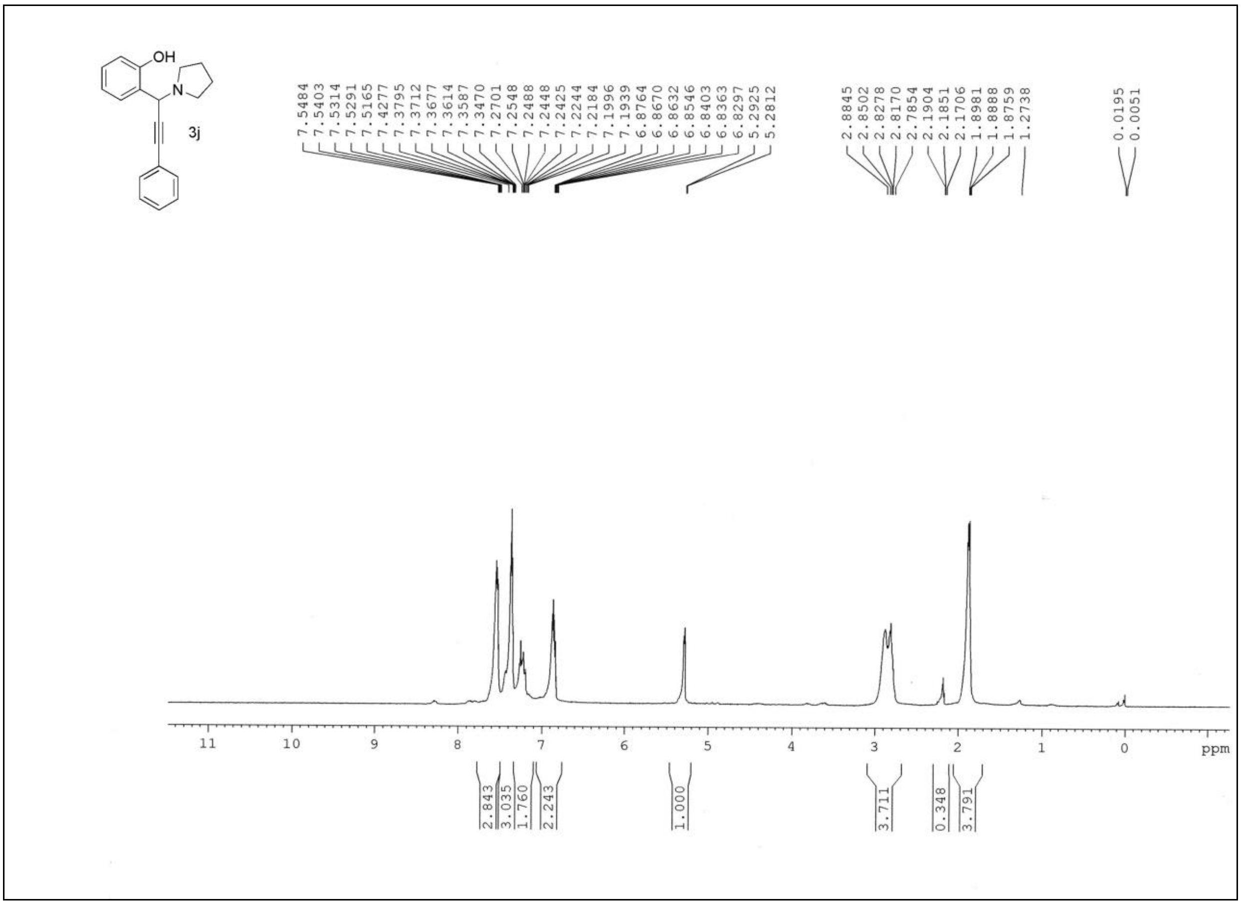
OH
N
3j
7.5484
7.5403
7.5314
7.5291
7.5165
7.4277
7.3795
7.3712
7.3677
7.3614
7.3587
7.3470
7.2701
7.2548
7.2488
7.2448
7.2425
7.2244
7.2184
7.1996
7.1939
6.8764
6.8670
6.8632
6.8546
6.8403
6.8363
6.8297
5.2925
5.2812
2.8845
2.8502
2.8278
2.8170
2.7854
2.1904
2.1851
2.1706
1.8981
1.8888
1.8759
1.2738
0.0195
0.0051
11
10
9
8
7
6
5
4
3
2
1
0
ppm
2.843
3.035
1.760
2.243
1.000
3.711
0.348
3.791

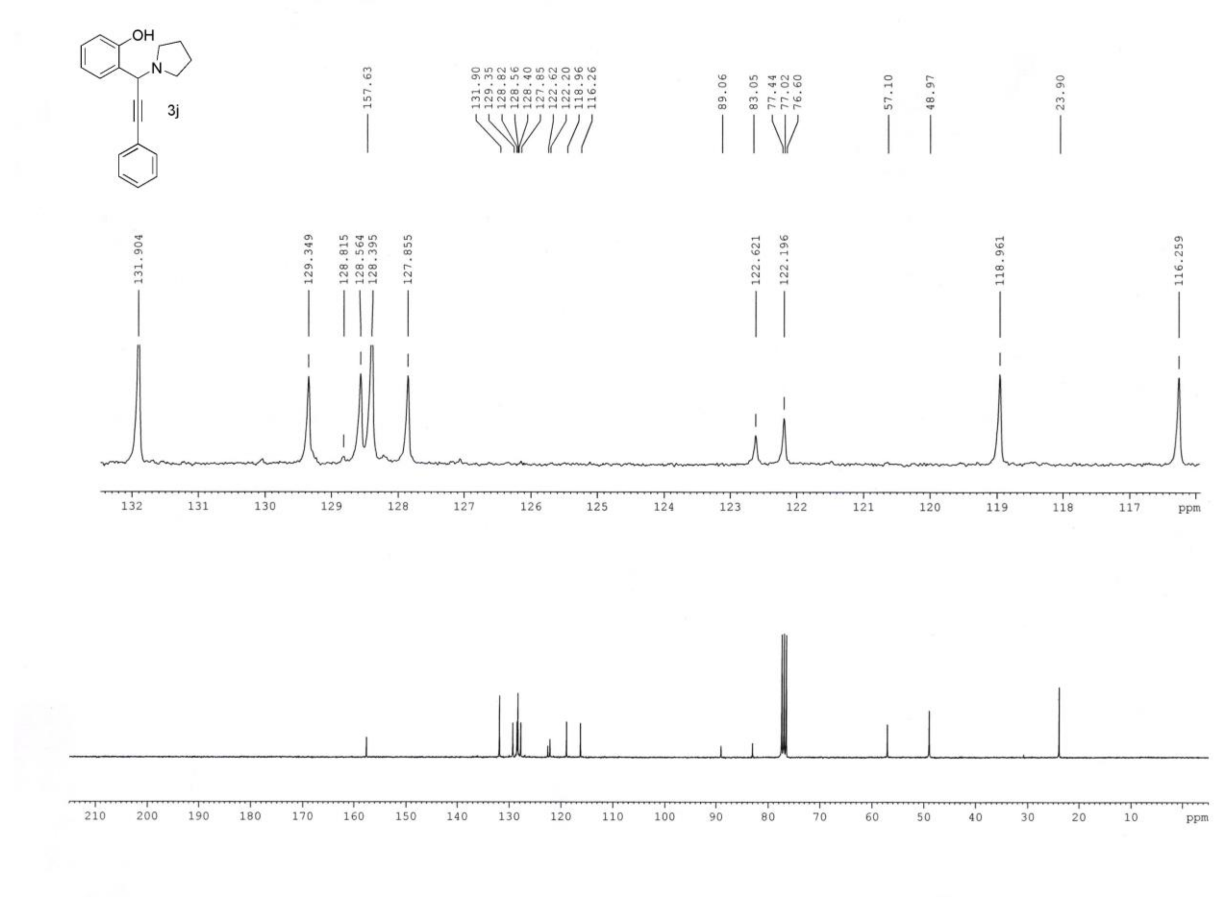
OH
N
3j
157.63
131.90
129.35
128.82
128.56
128.40
127.85
122.62
122.20
118.96
116.26
89.06
83.05
77.44
77.02
76.60
57.10
48.97
23.90
131.904
129.349
128.815
128.564
128.395
127.855
122.621
122.196
118.961
116.259
ppm
ppm

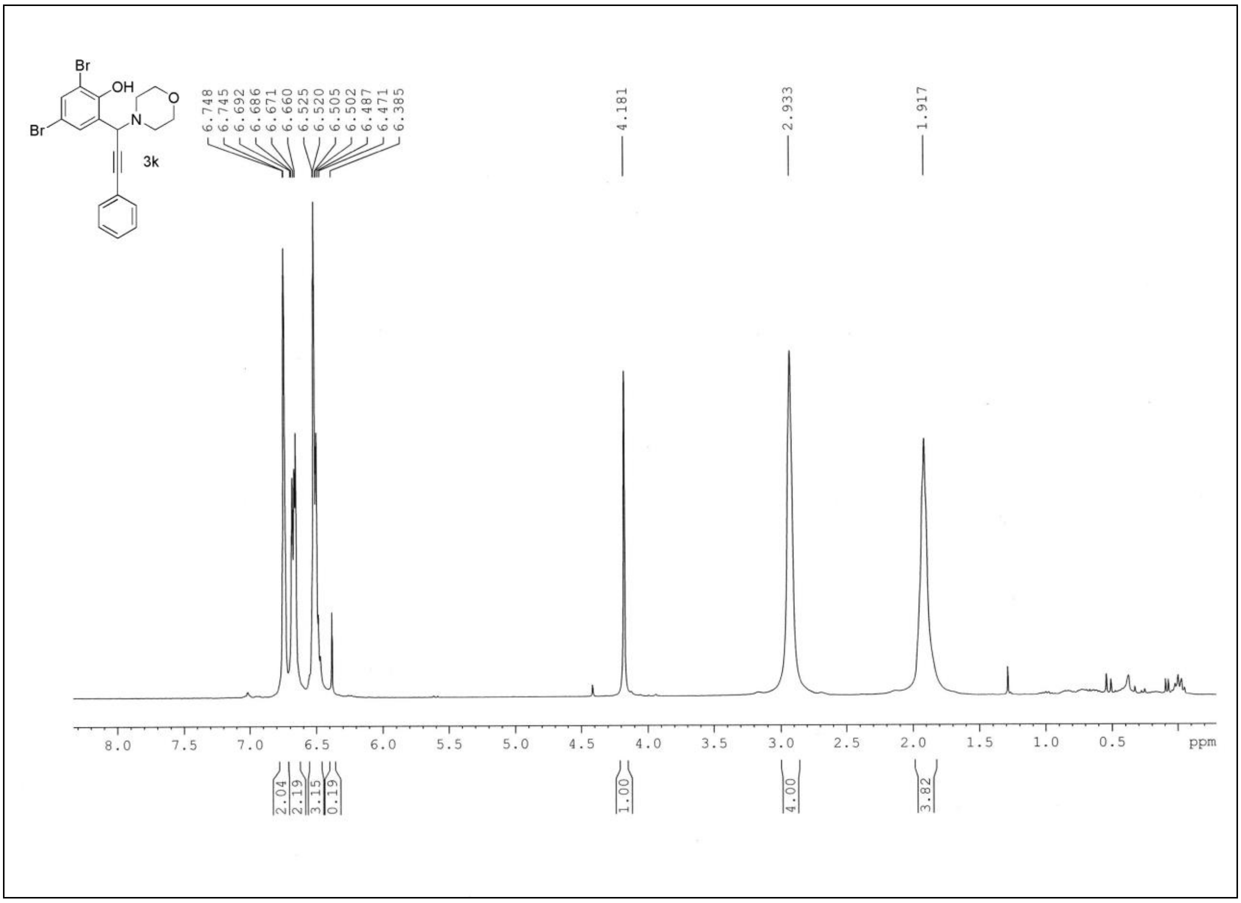

Br
OH
N
O
Br
3k
6.748
6.745
6.692
6.686
6.671
6.660
6.525
6.520
6.505
6.502
6.487
6.471
6.385
4.181
2.933
1.917
8.0 7.5 7.0 6.5 6.0 5.5 5.0 4.5 4.0 3.5 3.0 2.5 2.0 1.5 1.0 0.5 ppm
2.04
2.19
3.15
0.19
1.00
4.00
3.82

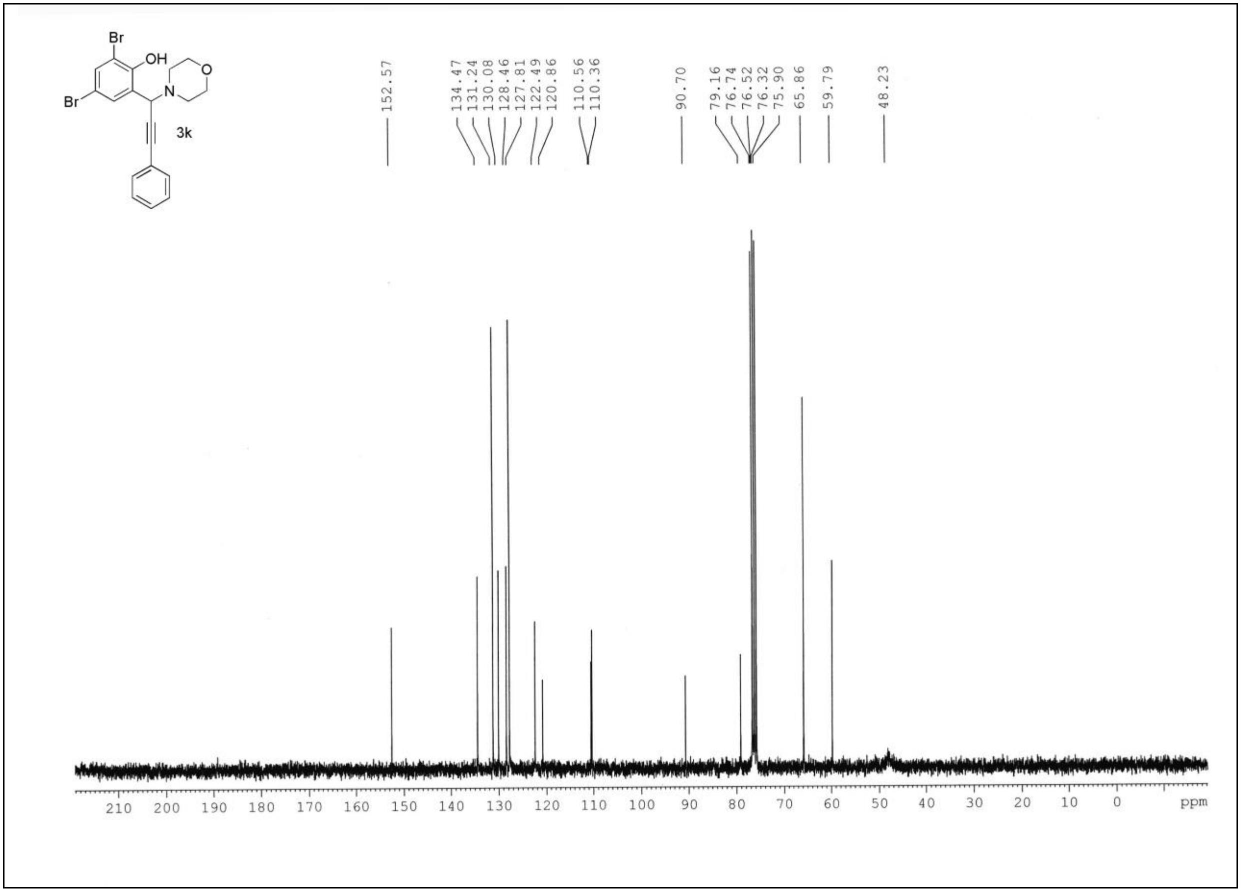

Br
OH
N
O
Br
3k
152.57
134.47
131.24
130.08
128.46
127.81
122.49
120.86
110.56
110.36
90.70
79.16
76.74
76.52
76.32
75.90
65.86
59.79
48.23
210 200 190 180 170 160 150 140 130 120 110 100 90 80 70 60 50 40 30 20 10 0 ppm

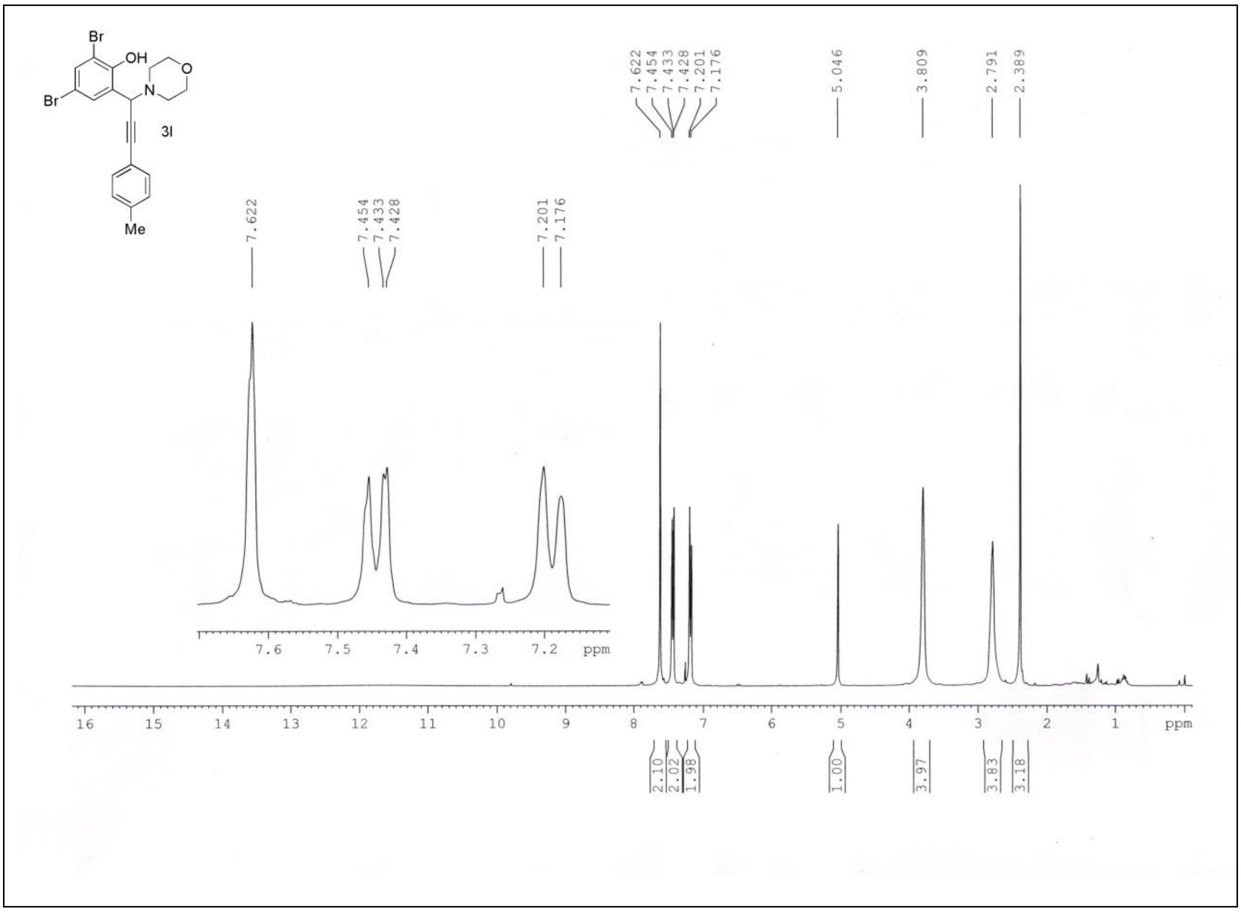

Br
OH
N
O
Br
3l
Me
7.622
7.454
7.433
7.428
7.201
7.176
5.046
3.809
2.791
2.389
7.6
7.5
7.4
7.3
7.2
ppm
16
15
14
13
12
11
10
9
8
7
6
5
4
3
2
1
ppm
2.10
2.02
1.98
1.00
3.97
3.83
3.18

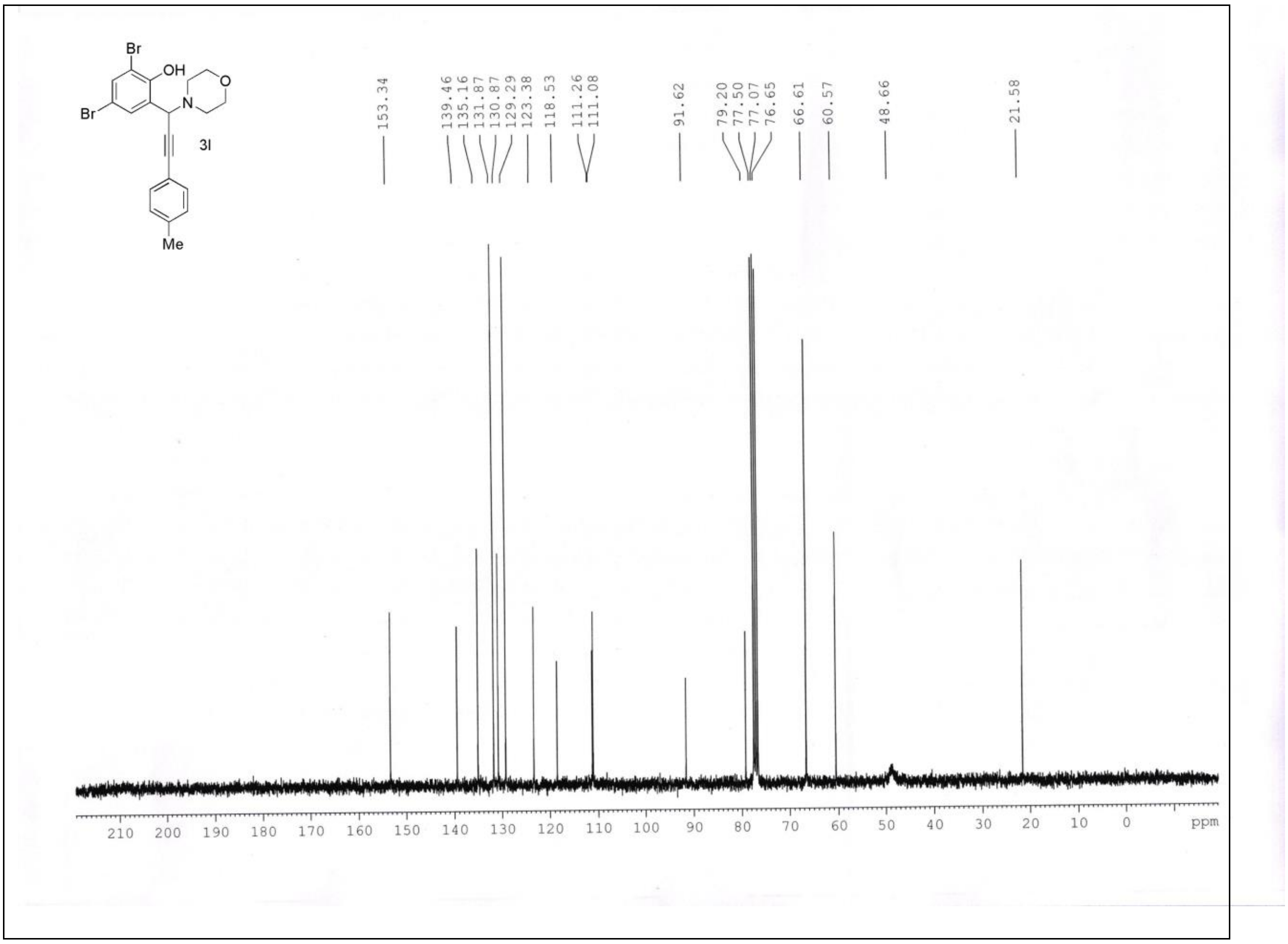

Br
OH
N
O
Br
3l
Me
153.34
139.46
135.16
131.87
130.87
129.29
123.38
118.53
111.26
111.08
91.62
79.20
77.50
77.07
76.65
66.61
60.57
48.66
21.58
210 200 190 180 170 160 150 140 130 120 110 100 90 80 70 60 50 40 30 20 10 0
ppm

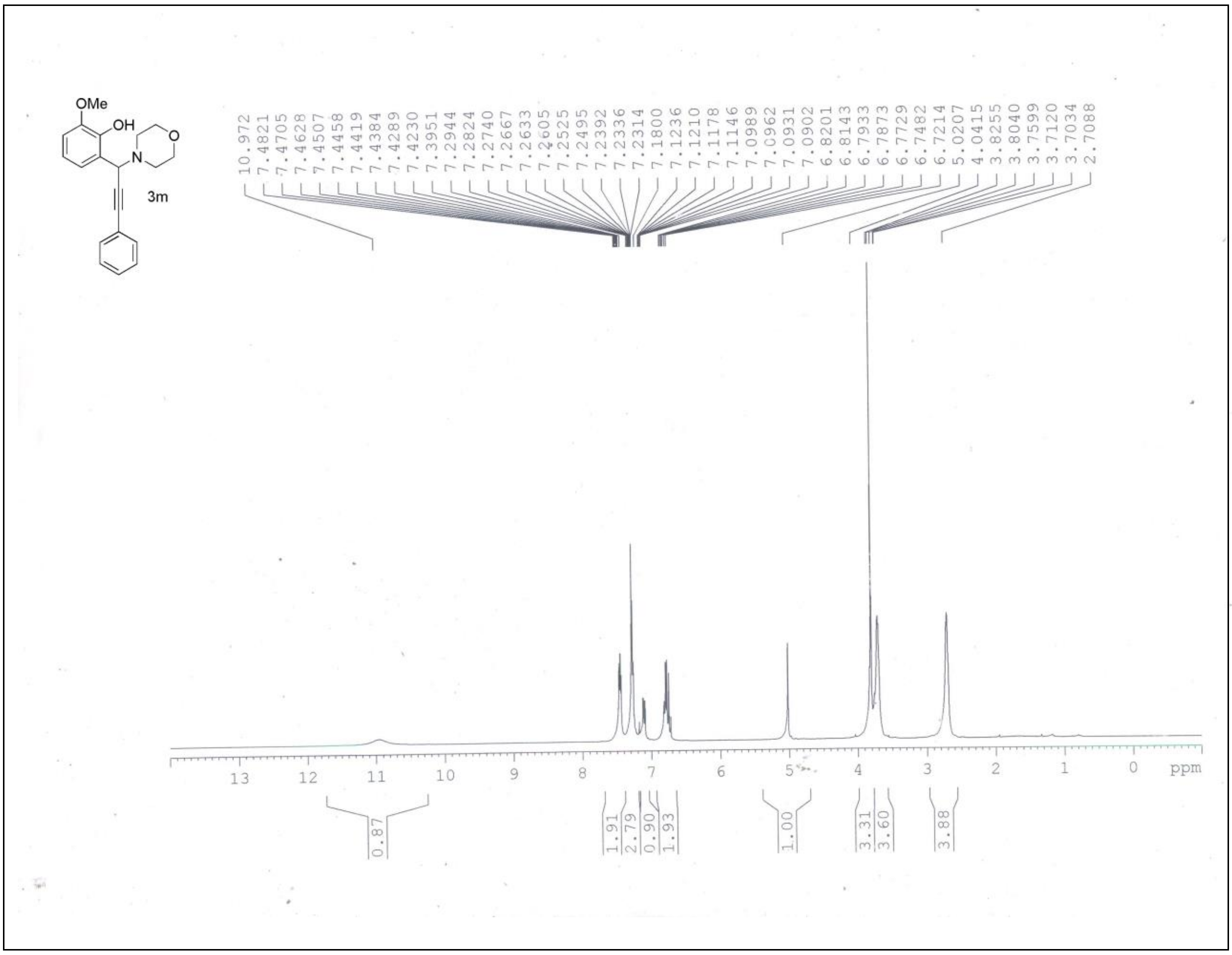
OMe
OH
N
O
3m
10.972
7.4821
7.4705
7.4628
7.4507
7.4458
7.4419
7.4384
7.4289
7.4230
7.3951
7.2944
7.2824
7.2740
7.2667
7.2633
7.2605
7.2525
7.2495
7.2392
7.2336
7.2314
7.1800
7.1236
7.1210
7.1178
7.1146
7.0989
7.0962
7.0931
7.0902
6.8201
6.8143
6.7933
6.7873
6.7729
6.7482
6.7214
5.0207
4.0415
3.8255
3.8040
3.7599
3.7120
3.7034
2.7088
13 12 11 10 9 8 7 6 5 4 3 2 1 0 ppm
0.87
1.91
2.79
0.90
1.93
1.00
3.31
3.60
3.88

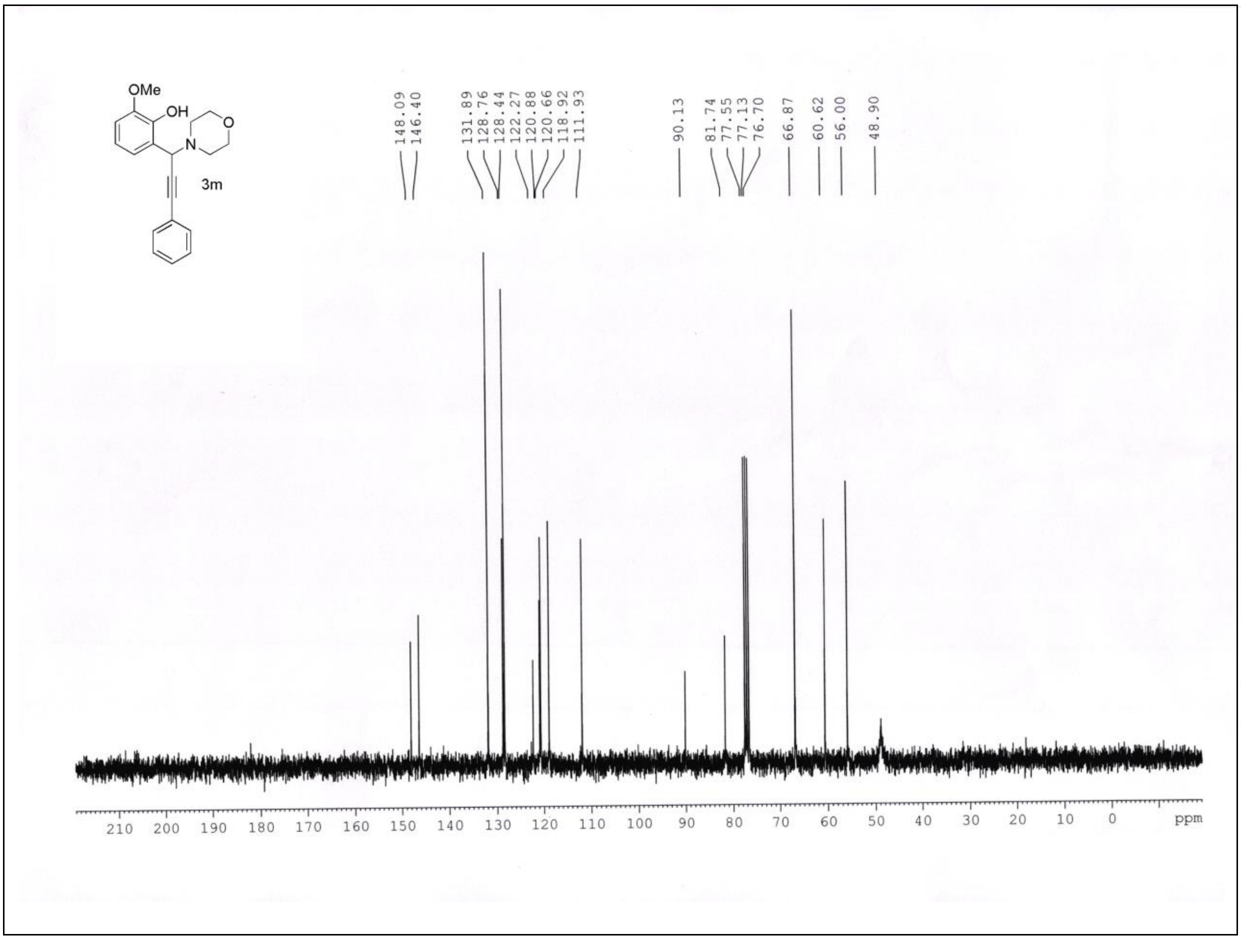

OMe
OH
N
O
3m
148.09
146.40
131.89
128.76
128.44
122.27
120.88
120.66
118.92
111.93
90.13
81.74
77.55
77.13
76.70
66.87
60.62
56.00
48.90
210 200 190 180 170 160 150 140 130 120 110 100 90 80 70 60 50 40 30 20 10 0 ppm

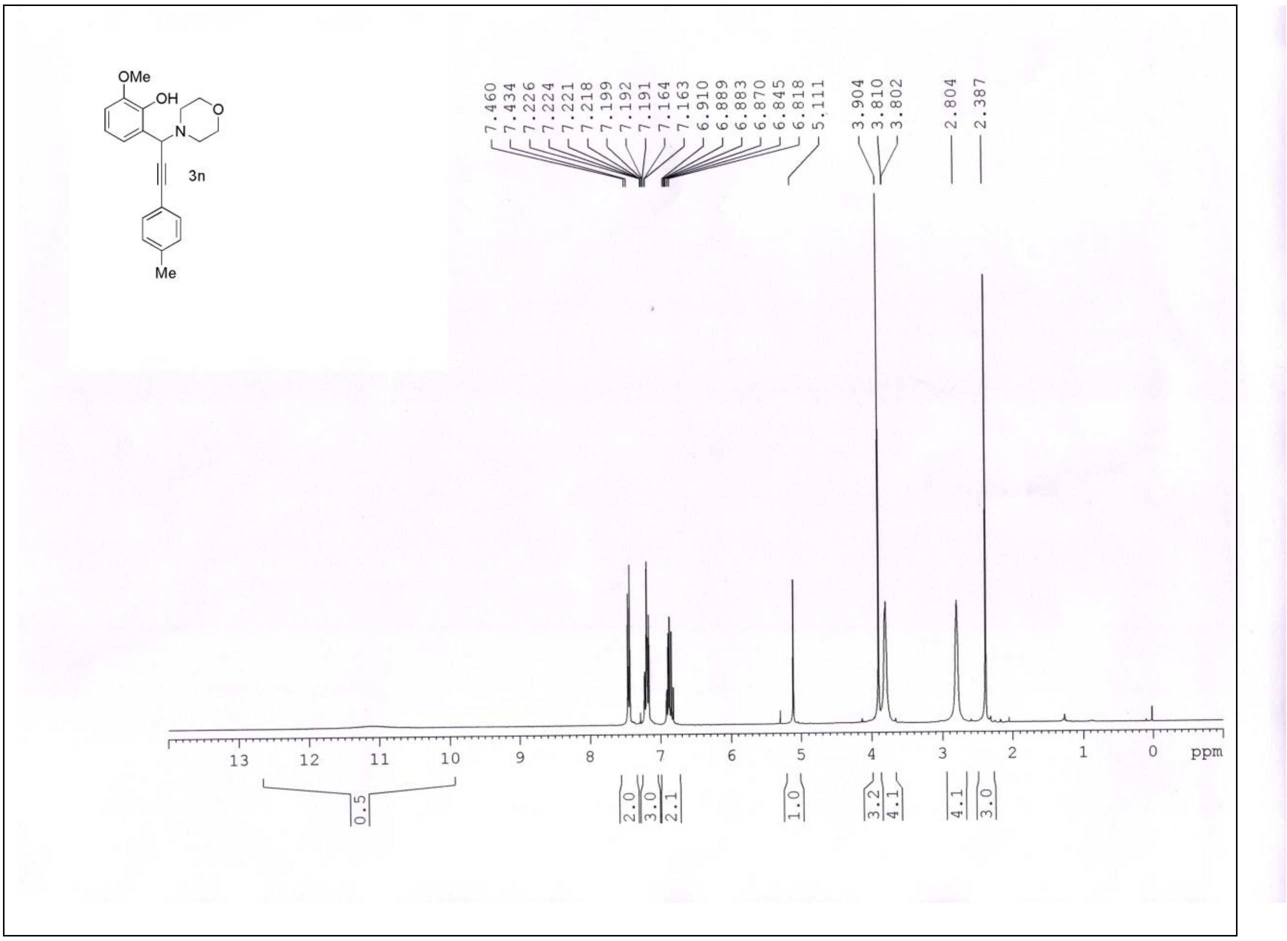
OMe
OH
N
O
3n
Me
7.460
7.434
7.226
7.224
7.221
7.218
7.199
7.192
7.191
7.164
7.163
6.910
6.889
6.883
6.870
6.845
6.818
5.111
3.904
3.810
3.802
2.804
2.387
13
12
11
10
9
8
7
6
5
4
3
2
1
0
ppm
0.5
2.0
3.0
2.1
1.0
3.2
4.1
4.1
3.0

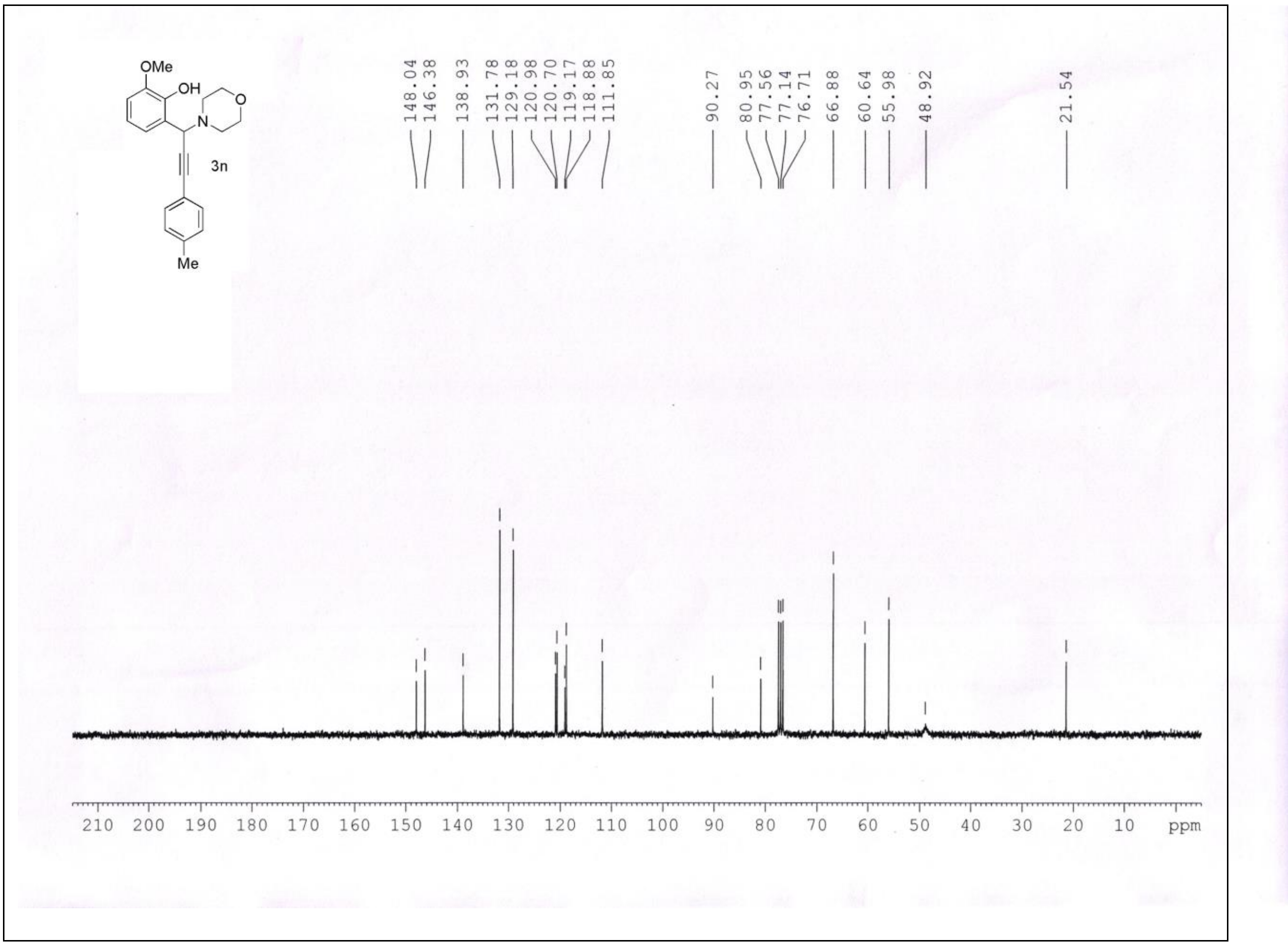
OMe
OH
N
O
3n
Me
148.04
146.38
138.93
131.78
129.18
120.98
120.70
119.17
118.88
111.85
90.27
80.95
77.56
77.14
76.71
66.88
60.64
55.98
48.92
21.54
210 200 190 180 170 160 150 140 130 120 110 100 90 80 70 60 50 40 30 20 10 ppm

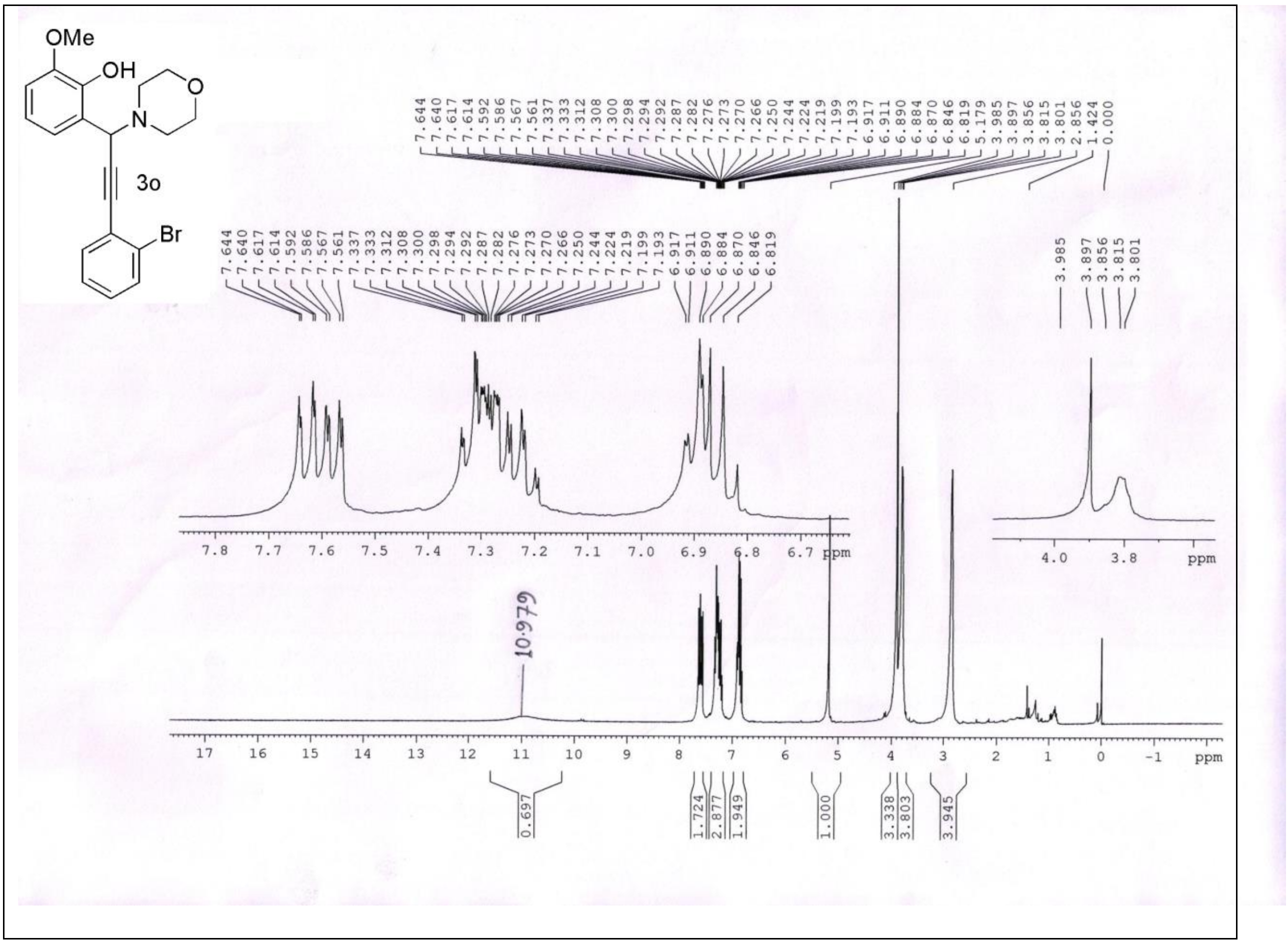
OMe
OH
N
O
3o
Br
10.979
ppm

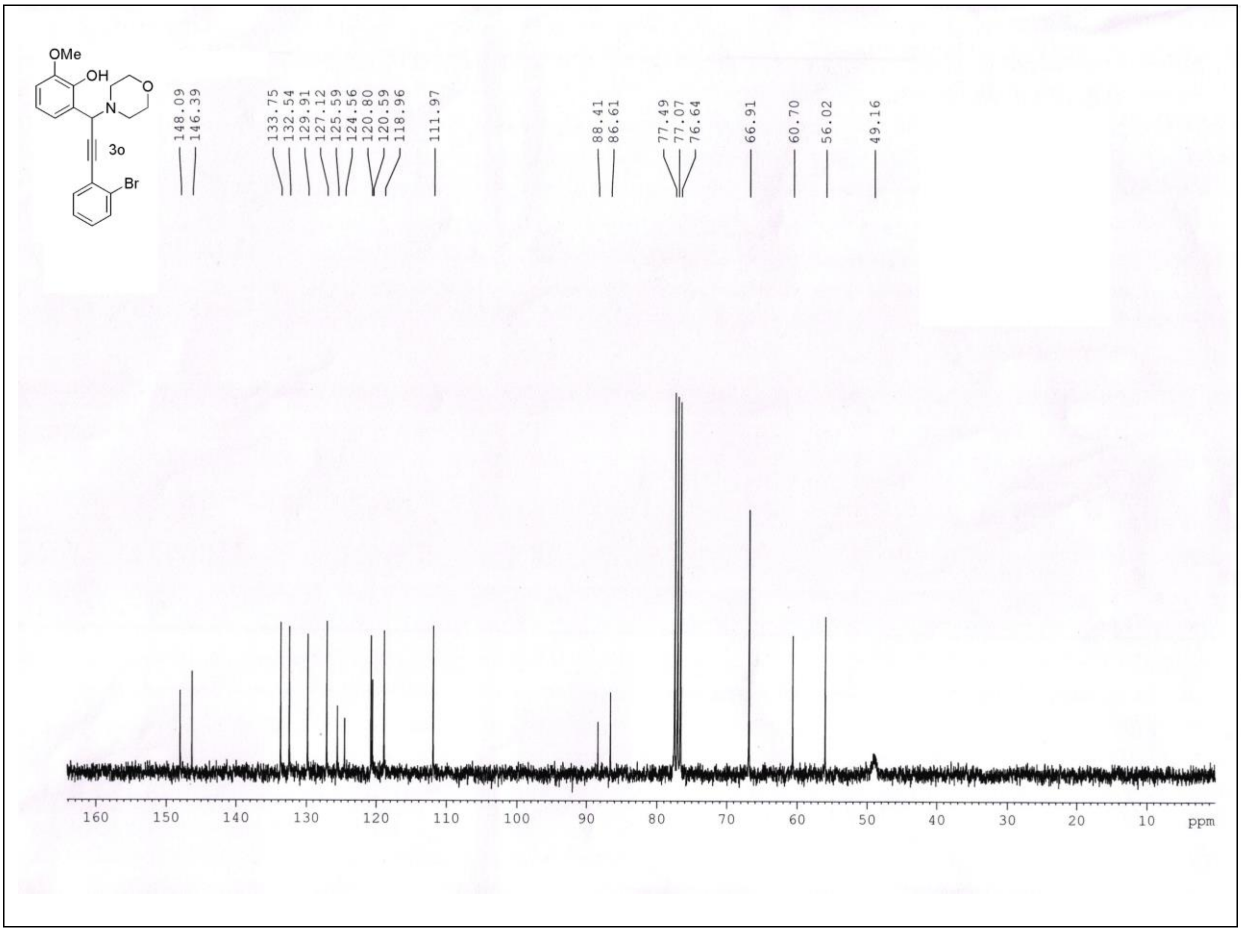
OMe
OH
N
O
3o
Br
148.09
146.39
133.75
132.54
129.91
127.12
125.59
124.56
120.80
120.59
118.96
111.97
88.41
86.61
77.49
77.07
76.64
66.91
60.70
56.02
49.16
160
150
140
130
120
110
100
90
80
70
60
50
40
30
20
10
ppm

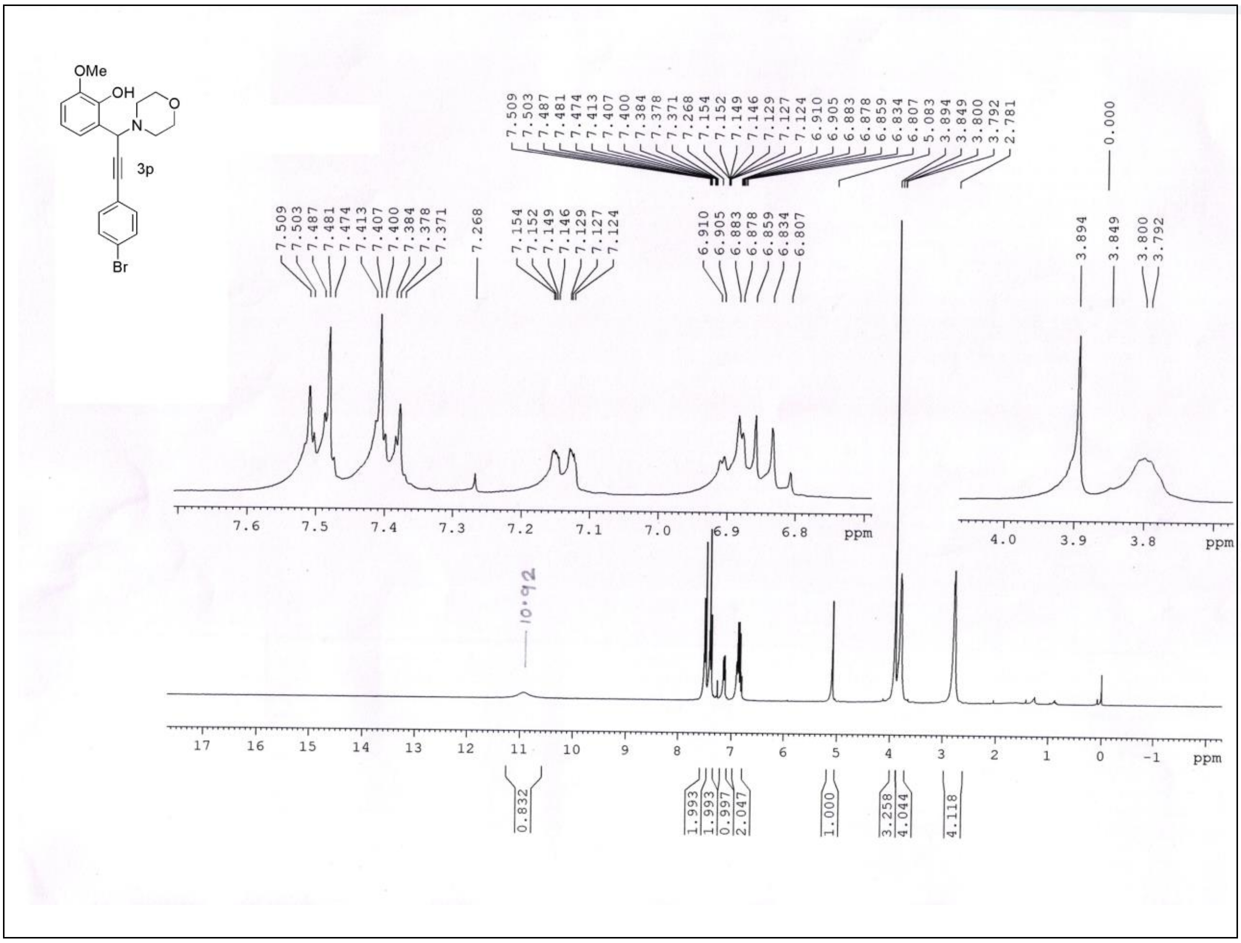

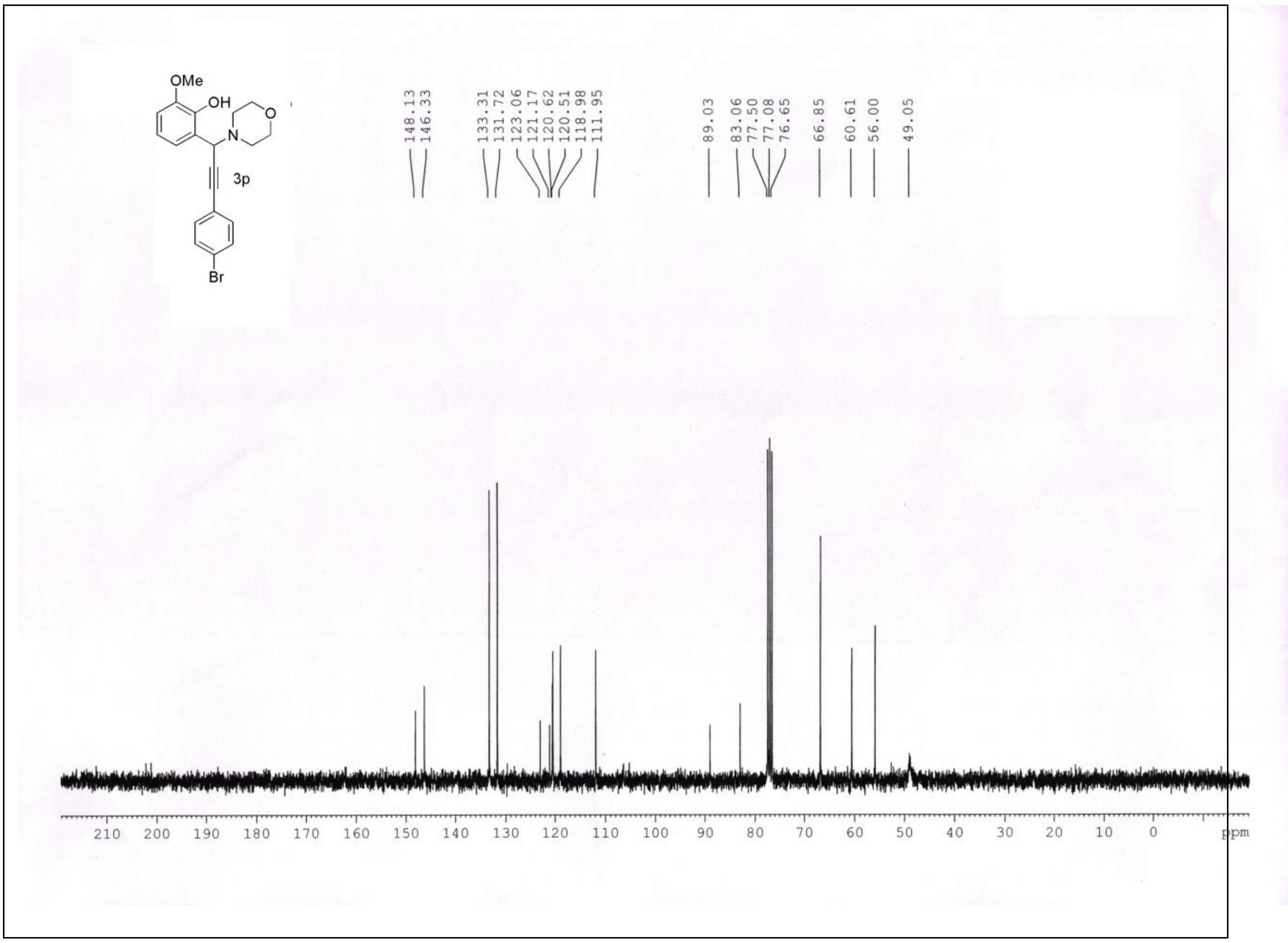
OMe
OH
N
O
3p
Br
148.13
146.33
133.31
131.72
123.06
121.17
120.62
120.51
118.98
111.95
89.03
83.06
77.50
77.08
76.65
66.85
60.61
56.00
49.05
210 200 190 180 170 160 150 140 130 120 110 100 90 80 70 60 50 40 30 20 10 0 ppm

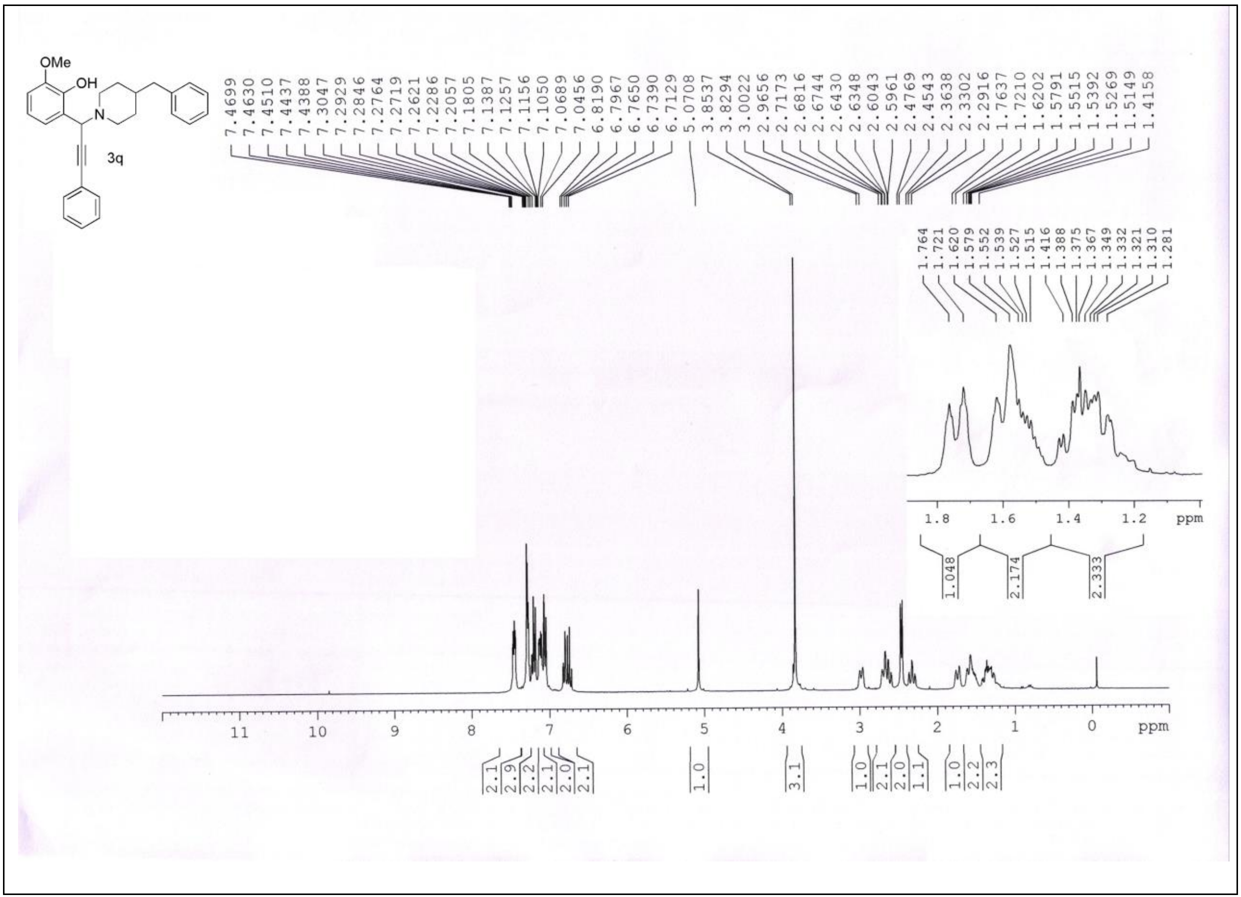
OMe
OH
N
3q

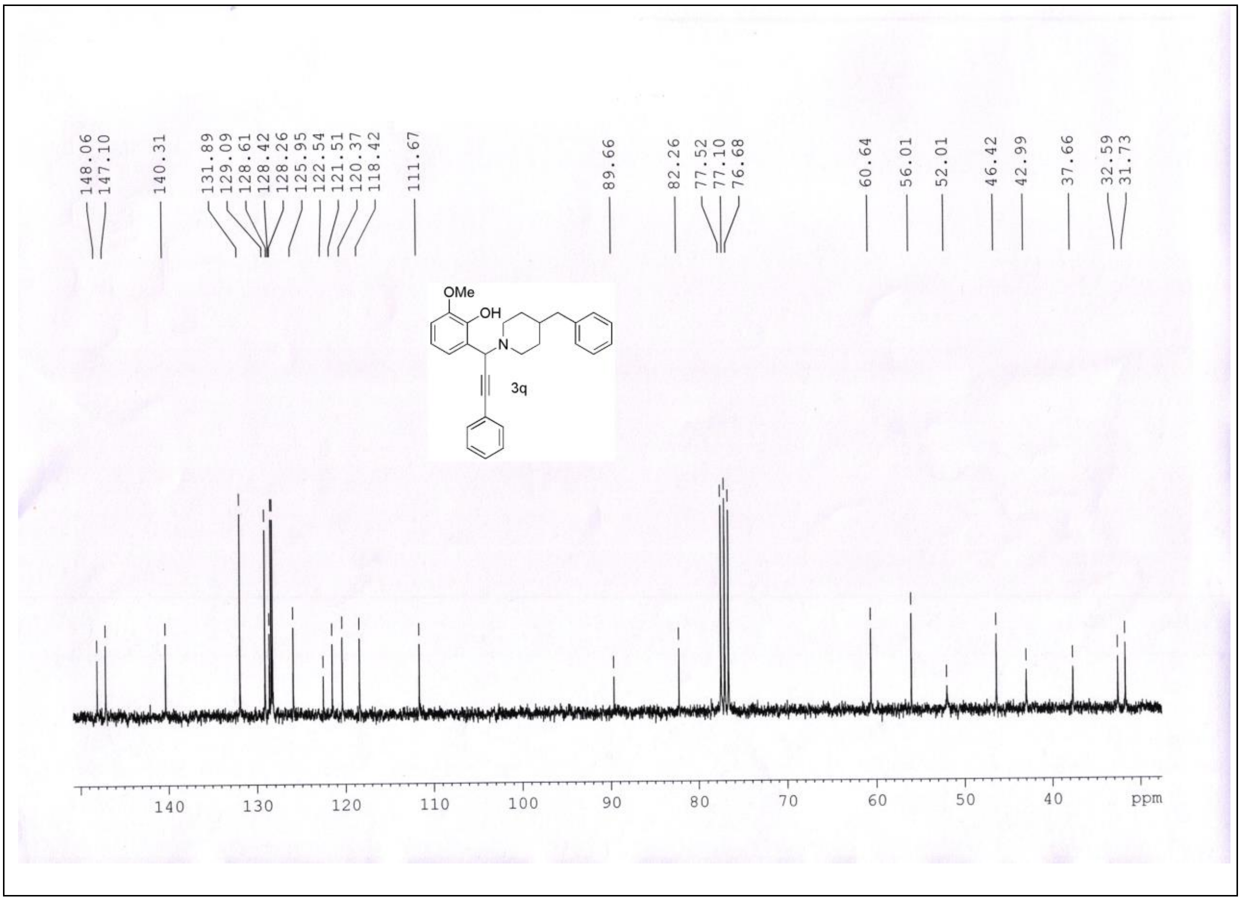
148.06
147.10
140.31
131.89
129.09
128.61
128.42
128.26
125.95
122.54
121.51
120.37
118.42
111.67
89.66
82.26
77.52
77.10
76.68
60.64
56.01
52.01
46.42
42.99
37.66
32.59
31.73
OMe
OH
N
3q
140
130
120
110
100
90
80
70
60
50
40
ppm

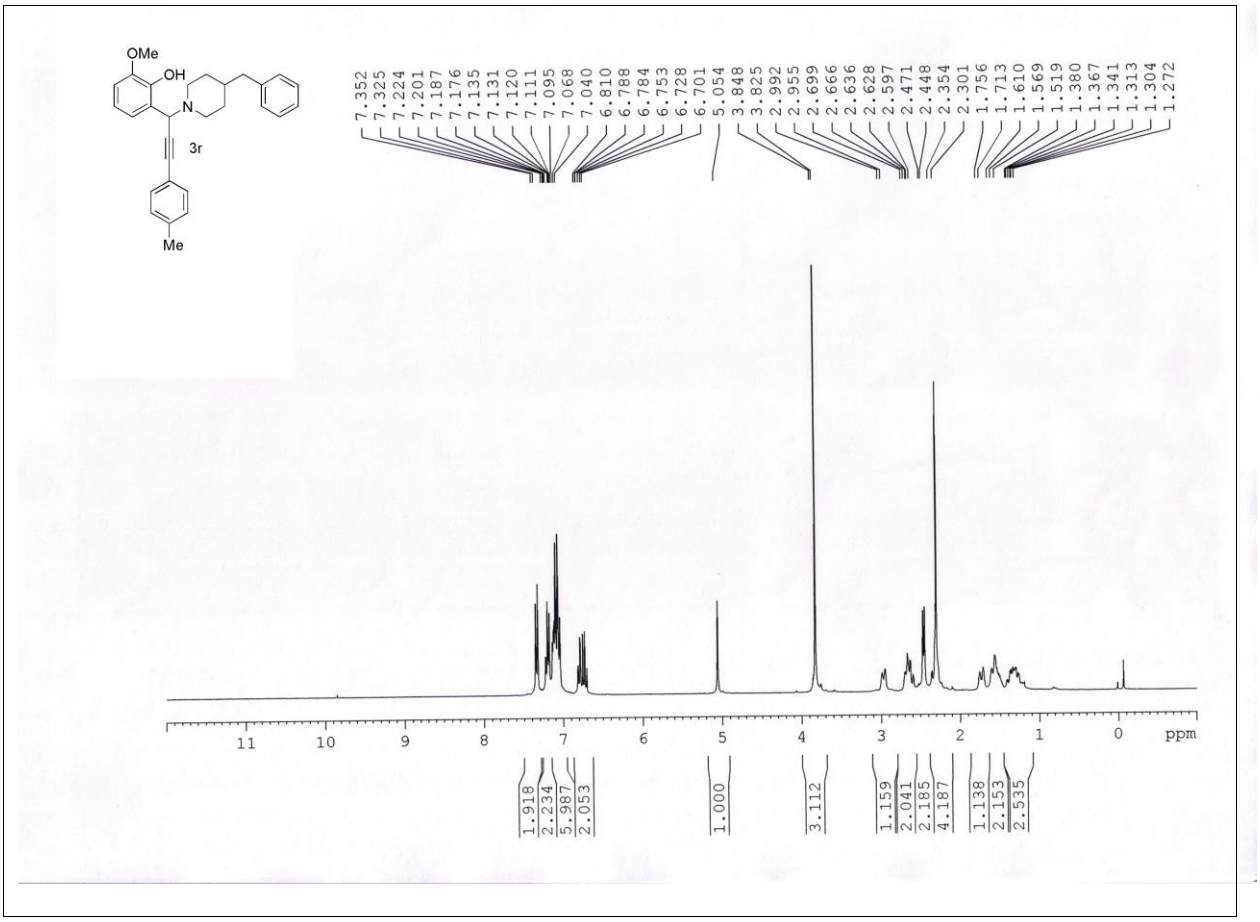
OMe
OH
N
3r
Me
7.352
7.325
7.224
7.201
7.187
7.176
7.135
7.131
7.120
7.111
7.095
7.068
7.040
6.810
6.788
6.784
6.753
6.728
6.701
5.054
3.848
3.825
2.992
2.955
2.699
2.666
2.636
2.628
2.597
2.471
2.448
2.354
2.301
1.756
1.713
1.610
1.569
1.519
1.380
1.367
1.341
1.313
1.304
1.272
11
10
9
8
7
6
5
4
3
2
1
0
ppm
1.918
2.234
5.987
2.053
1.000
3.112
1.159
2.041
2.185
4.187
1.138
2.153
2.535

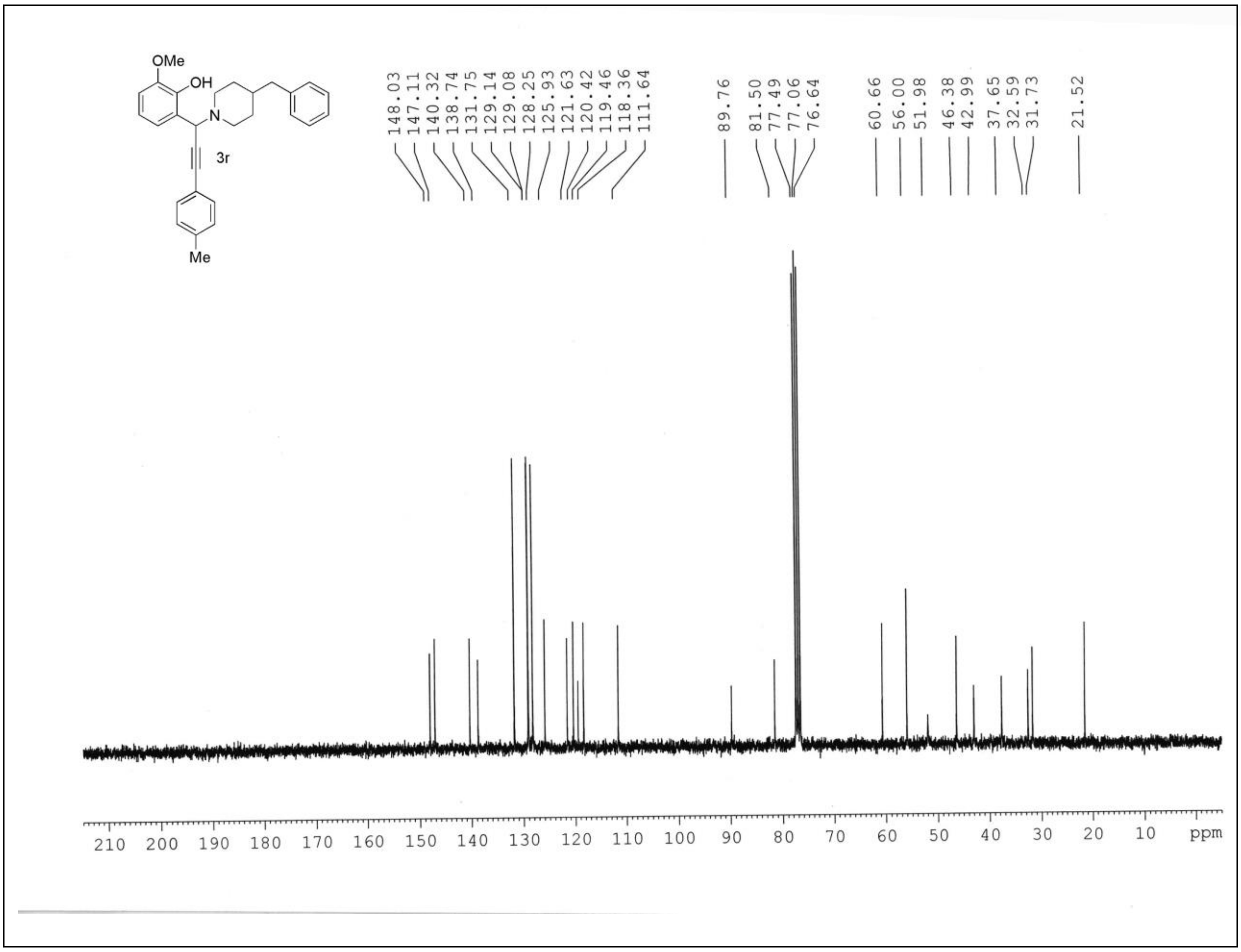
OMe
OH
N
3r
Me
148.03
147.11
140.32
138.74
131.75
129.14
129.08
128.25
125.93
121.63
120.42
119.46
118.36
111.64
89.76
81.50
77.49
77.06
76.64
60.66
56.00
51.98
46.38
42.99
37.65
32.59
31.73
21.52
210 200 190 180 170 160 150 140 130 120 110 100 90 80 70 60 50 40 30 20 10 ppm

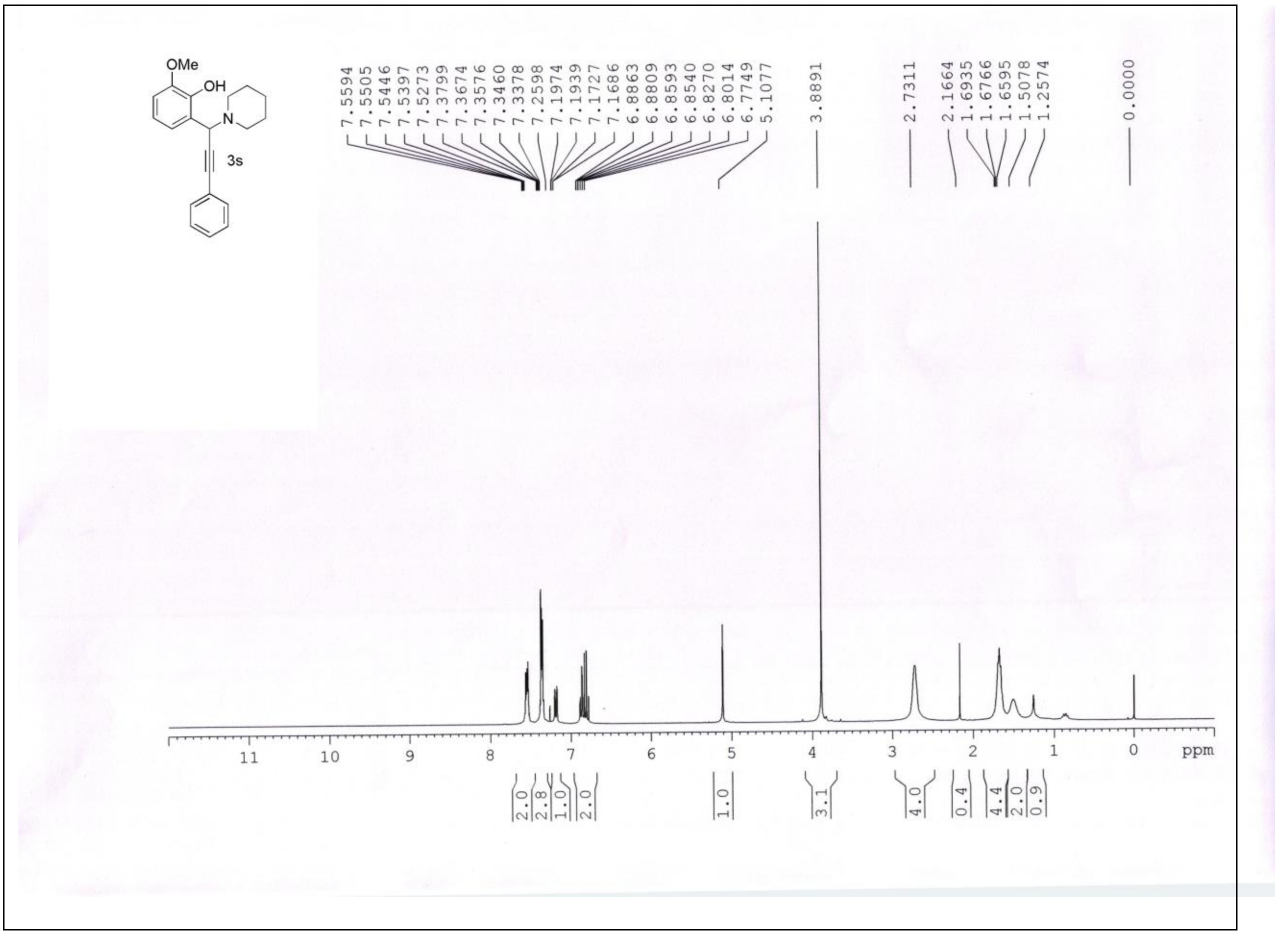
OMe
OH
N
3s
7.5594
7.5505
7.5446
7.5397
7.5273
7.3799
7.3674
7.3576
7.3460
7.3378
7.2598
7.1974
7.1939
7.1727
7.1686
6.8863
6.8809
6.8593
6.8540
6.8270
6.8014
6.7749
5.1077
3.8891
2.7311
2.1664
1.6935
1.6766
1.6595
1.5078
1.2574
0.0000
11
10
9
8
7
6
5
4
3
2
1
0
ppm
2.0
2.8
1.0
2.0
1.0
3.1
4.0
0.4
4.4
2.0
0.9

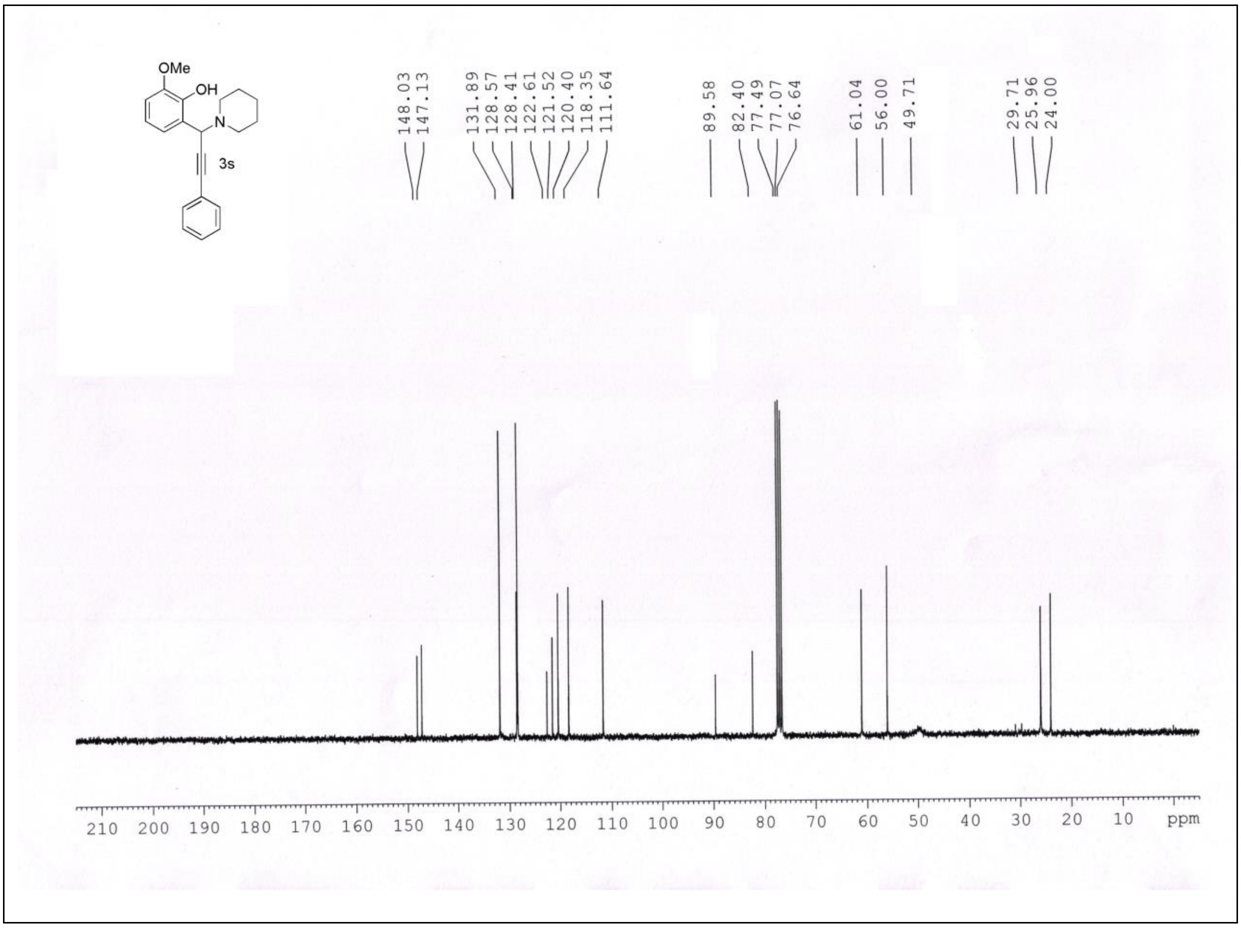
OMe
OH
N
3s
148.03
147.13
131.89
128.57
128.41
122.61
121.52
120.40
118.35
111.64
89.58
82.40
77.49
77.07
76.64
61.04
56.00
49.71
29.71
25.96
24.00
210 200 190 180 170 160 150 140 130 120 110 100 90 80 70 60 50 40 30 20 10 ppm

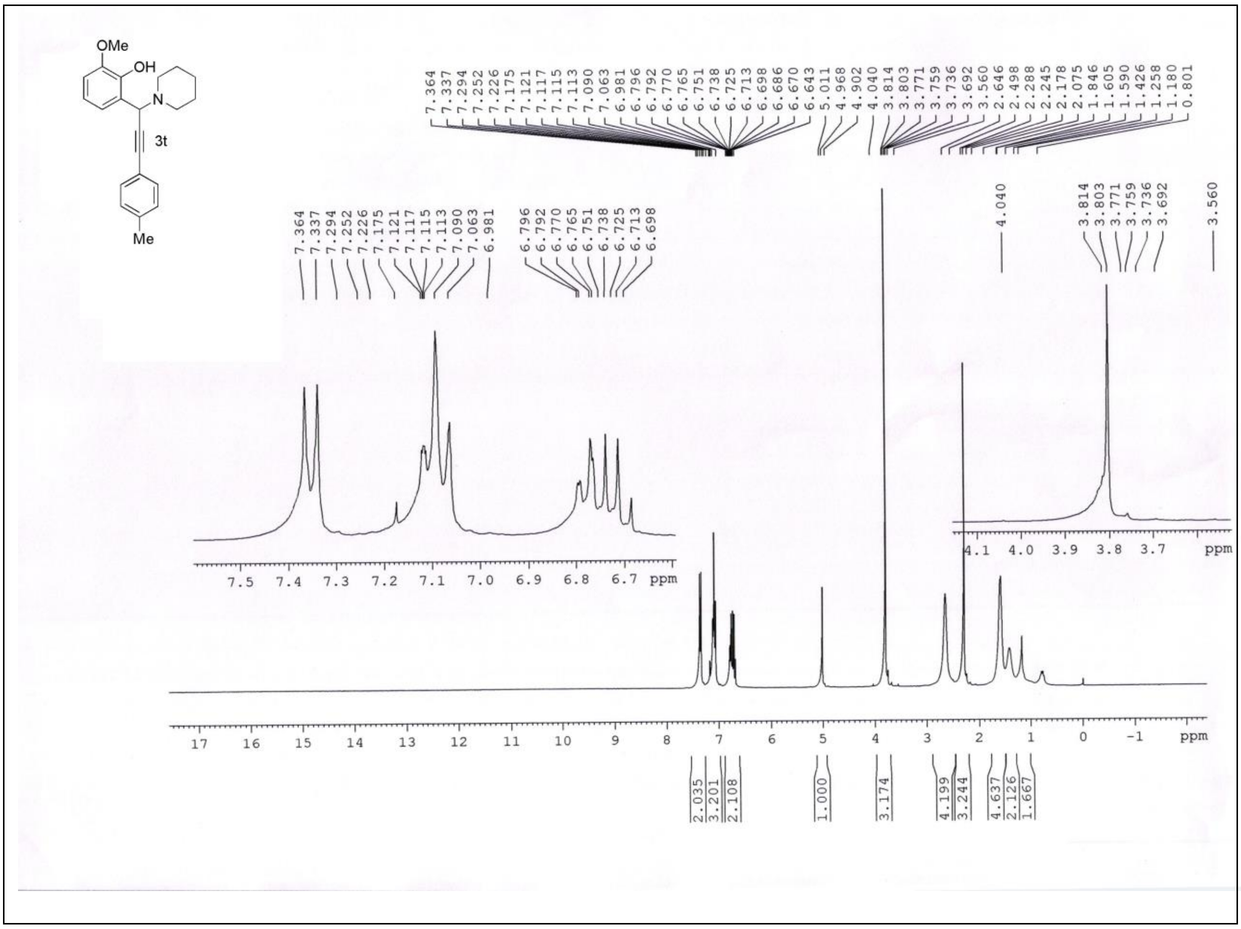

OMe
OH
N
3t
Me
ppm

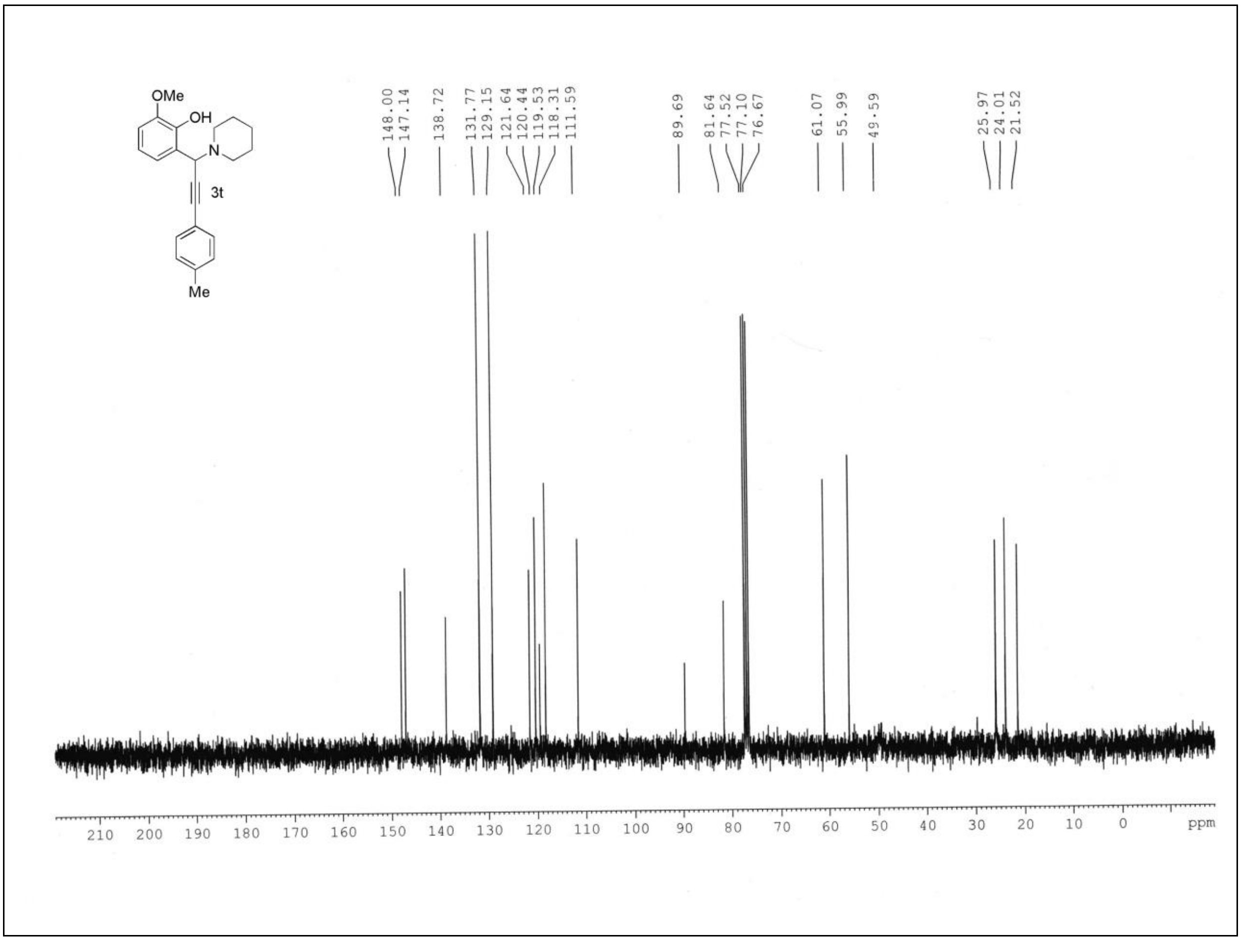
OMe
OH
N
3t
Me
148.00
147.14
138.72
131.77
129.15
121.64
120.44
119.53
118.31
111.59
89.69
81.64
77.52
77.10
76.67
61.07
55.99
49.59
25.97
24.01
21.52
210 200 190 180 170 160 150 140 130 120 110 100 90 80 70 60 50 40 30 20 10 0 ppm
